# Rational modulation of immune mechanisms synergizes the anti-tumor effects of targeted radiation therapy in pre-clinical models

**DOI:** 10.3389/fimmu.2026.1637129

**Published:** 2026-03-27

**Authors:** Tristan Wirtz, Catherine Lee, Sripad Ram, Tao Xie, Sepideh Mojtahedzadeh, Nicole Streiner, Kavon Noorbehesht, Lisa K. Manzuk, Allison Rohner, Edward Cabral, Vinicius Bonato, Timothy Affolter, Manfred Kraus, Christopher Dillon, Anand Giddabasappa

**Affiliations:** 1 Oncology R&D, Pfizer Inc, La Jolla, CA, United States; 2 Animal Models and Imaging, Comparative Medicine-Drug Safety R&D, Pfizer Inc, La Jolla, CA, United States; 3 Global Pathology, Comparative Medicine-Drug Safety R&D, Pfizer Inc, La Jolla, CA, United States; 4 Non-Clinical Statistics, Worldwide Research & Development (WRD), Pfizer Inc, La Jolla, CA, United States

**Keywords:** combination therapy, tumor-infiltrating immune cells, immuno-oncology, macrophages, synergism, T cells, targeted radiation therapy, tumor models

## Abstract

Immunotherapy has revolutionized cancer treatment, offering new hope for many patients. However, while some individuals show remarkable responses, the overall success rate remains limited. This has spurred interest in combination therapies, particularly with established treatments like radiation therapy (RT), to improve outcomes. RT is a cornerstone of cancer therapy and known to influence the immune landscape, yet a systematic characterization of its effects on tumor-infiltrating leukocytes (TILs) and a rationale-based therapy is still lacking. In this study, we employed a diverse set of pre-clinical syngeneic murine tumor models with varying immune profiles to investigate the immunological impact of tumor targeted RT. We observed that immunologically ‘hot’ tumors showed stronger tumor growth inhibition (TGI) after RT compared to ‘cold’ tumors. Additionally, RT induced both pro- and anti-inflammatory shifts within the tumor immune microenvironment. Importantly, RT led to an intra-tumoral increase in proliferating CD8^+^ T cells, while the population of proliferating macrophages was notably reduced. To identify immune-modulatory pathways that shape the response to RT across different tumor immune contexts, we tested RT in HPK1 (Hematopoietic Progenitor Kinase 1) and STING (Stimulator of Interferon Genes) deficient mice. These experiments revealed that STING deficiency compromises TGI in tumors with a high baseline population of myeloid cells expressing an interferon response signature. Moreover, we identified a synergistic effect on survival in tumor-bearing mice when combining HPK1 deficiency with RT. Thus, RT promotes expansion of cytotoxic T cells while limiting macrophage proliferation, with therapeutic outcomes strongly influenced by STING and HPK1 pathways. Collectively, these results highlight the complex interplay between RT, tumor immune microenvironment and response to therapy, offering potential avenues for novel therapeutic combinations.

## 1 Introduction

Cancer is a leading cause of death worldwide, with nearly 20 million new cases and 9.7 million deaths reported in 2022 (1). In the United States, it remains the second leading cause of death (2). Radiation therapy (RT) has been used in cancer treatment for many decades (3). Advances in radiation technologies, such as stereotactic body radiation therapy now allow tumors to be treated with fewer, higher doses and increased precision, thereby sparing adjacent tissues (4, 5). Today, approximately 60% of patients undergo RT at some point during their treatment (6, 7).

Historically, RT was primarily used to induce tumor cell death (8). However, with the advent of checkpoint inhibitors (CPI), the critical role of the immune system in controlling tumor growth has become increasingly recognized (9). CPI treatments can result in strong and lasting tumor growth inhibition (TGI), but effectiveness varies between tumor types, with only about 25% of patients experiencing long-term tumor regression (10, 11). Consequently, combination therapies are being explored as a promising approach to enhance anti-tumor immune responses in more patients (12, 13). Among potential partners for immunotherapy, RT is particularly promising due to its ability to induce an immunogenic cell death (ICD) (14).

RT leads to the release of tumor-associated neo-antigens and inflammatory signals, induces T-cell-mediated ICD, and activates dendritic cells (DCs) for cross-priming (15). These processes alter the tumor microenvironment (TME), increase mutational burden, expand the T-cell receptor (TCR) repertoire, induce major histocompatibility complex-1 (MHC-I) expression on tumor cells, and initiate local and systemic ‘danger’ responses, potentially leading to regression of primary and secondary tumors, a phenomenon known as the “abscopal effect” (16). Unfortunately, RT as a monotherapy has not achieved durable anti-tumor immune responses, because it also recruits immunosuppressive cells and upregulates checkpoint proteins on tumor cells, enabling immune evasion.

Recent insights into the effects of RT across various tumor types and preclinical models highlight the need to adapt treatment regimens to biological and clinical contexts to achieve optimal outcomes (17). The impact of RT on the TME varies depending on factors such as radiation dose, fractionation regimen, tumor type, and immune cell infiltration at the start of treatment. For instance, a preclinical study demonstrated that fractionated RT, but not single-dose RT, induces an abscopal effect when combined with anti-CTLA-4 (Cytotoxic T-lymphocyte-associated protein 4) therapy (18). This difference arises because higher single-dose RT activates the DNA exonuclease Trex1 (Three Prime Repair Exonuclease 1), which suppresses the activation of the cyclic GMP-AMP synthase - stimulator of interferon genes (cGAS-STING) (19). However, despite these insights, there remains a lack of systematic evaluation of the effects of RT in tumor models of different immune phenotypes.

RT induces profound changes in the TME with a substantial influence on the tumor infiltrating immune cells. Immunological effects of RT are both pro- and anti-inflammatory with an overall trend towards immune activation (8, 19). So far, the effects of RT in combination with clinically available CPIs have fallen short of expectations (20). Although initial case reports suggested strong synergistic anti-tumor effects of RT combined with immunotherapy (12, 21). Various combinations of immunotherapy and RT were effective in pre-clinical animal models (22). However, most studies focused on either a specific cancer indication or immunotherapeutic. As a result, systematic analysis of RT effects on immune cell infiltration across tumors with different immune profiles are still missing. Therefore, a better understanding of the immunological effects of RT for rationalistic combinations with approved or possibly novel immune therapy targets are necessary. In this study, we systematically evaluated the effects of RT across tumor models of various immune-phenotypes. RT altered multiple immune cell populations, especially CD8^+^ (Cluster of Differentiation 8) T cells, NK (Natural Killer) cells and myeloid cells. STING and HPK1 are known regulators of myeloid and CD8^+^ T cell activation and function (23–28), and their modulation is currently being explored as a therapeutic strategy (29, 30). To illustrate a rationale-driven evaluation of pathways influencing these key immune populations, we focused on STING and HPK1, given their clinical relevance.

## 2 Methods

### 2.1 Animal studies

All animal protocols were reviewed and approved by the Pfizer Global Research and Development Institutional Animal Care and Use Committee (IACUC). C57BL/6 mice, as well as STING Golden ticket mice (C57BL/6J-Sting1^gt^/J) were procured from The Jackson Laboratory (Bar Harbor, ME) and BALB/c mice were procured from Charles River Laboratories (San Diego, CA).

HPK1-deficient mice (HPK1^-/-^) were generated by flanking the region encompassing exon 6 to exon 9 of the Map4K1 gene with LoxP sites in the same orientation and then crossing these mice with a Cre deleter mouse strain (C57BL/6N-Gt(ROSA)26Sor^tm1(cre)^) and then bred to homozygosity sourced from The Jackson Laboratory. All studies were performed in animal rooms which were temperature (20 – 26 °C) and humidity (30 – 70 %) controlled. The animals were under a 12 h:12 h light-dark cycle and had *ad libitum* access to water and Laboratory Rodent Diet. Animals had at least 3 days of acclimation prior to study initiation.

### 2.2 Cell culture

All murine cancer cell lines were cultured at 37 °C and 5% CO_2_. CT26 (colon carcinoma), B16F10 (melanoma), EMT6 (breast cancer) were obtained from the American Type Culture Collection (ATCC, Manassas, VA); MC38-Kerafast cells were obtained from Kerafast (Boston, MA) and all cell lines were cultured as per manufacturer’s instructions. MC38-Ribas cells were obtained from Antoni Ribas lab cultured in DMEM supplied with 2 mM l-glutamine, 10% FBS, penicillin (100 U/ml) and streptomycin (100 μg/ml) (Thermo Fisher Scientific, Waltham, MA). All cell lines were maintained by Pfizer Oncology Cell Bank and were validated using standard microbial and short tandem repeat (STR) profiling tests at IDEXX BioAnalytics (Columbia, MO).

### 2.3 *In vitro* cell growth assays

Cells were detached using 0.25% Trypsin-EDTA (Thermo Fisher, Waltham, MA) and washed and resuspended in growth medium. After counting, cells were irradiated using X-Rad 225 (see below) and then seeded in 12-well plates. For EMT6 cells 1×10^4^ cells were seeded, for all other cell lines 2.5×10^4^ cells were seeded per well in a 12-well plate. Cell proliferation was monitored using the Incucyte^®^ live-cell analysis system (Sartorius AG, Göttingen, Germany). Phase-contrast images were acquired at regular intervals, and cell confluence was quantified as the percentage of the culture surface area occupied by cells using the manufacturer’s integrated image-analysis software.

### 2.4 Tumor targeted RT

For RT a X-Rad 225 biological irradiator (Precision X-Ray, Madison, CT) was used at a dose output of 225 kV, 10 mA. Cells were irradiated using a 2 mm Aluminum filter and targeted irradiation of tumor bearing mice was performed using a 0.3 mm Copper filter with a 5 × 5 mm collimator. In the study with two-fractionation arm, RT was delivered using the same setup and dose rate with 24 h of separation between each fraction.

### 2.5 *In vivo* syngeneic tumor models

For *in vivo* syngeneic tumor models, 5 × 10^5^ MC38 cells or 2 × 10^5^ B16F10 cells were subcutaneously implanted on the right flanks of 8- to 10-week-old female C57BL/6 mice. EMT6 cells (2.5 × 10^5^) or CT26 cells (2.5 × 10^5^) were implanted on the right flanks of 8- to 10-week-old female BALB/c mice. 5 × 10^5^ MC38 cells were implanted in STING-Golden ticket and HPK1-deficient mice.

Tumors were measured at least twice weekly using a caliper on the longest dimension (length) and the longest perpendicular dimension (width). Tumor volume was estimated with the formula: (L × W²)/2. Animals were randomized and enrolled into treatment arms when tumors were between 75–150 mm³. Mice with tumor volumes greater than 2000 mm³ were euthanized by CO_2_ inhalation.

### 2.6 Flow cytometry and scRNA-seq

To obtain single-cell suspensions, tumors were processed using the Tumor Dissociation Kit, mouse (Miltenyi Biotec, Bergisch Gladbach, Germany) according to the manufacturer’s instructions. Following dissociation, cells were resuspended in autoMACS^®^ Rinsing Solution with MACS^®^ BSA Stock Solution diluted 1:20 and the cell suspensions were passed through a 70-µm cell strainer.

Single cell suspensions were obtained from mouse spleen and lymph node samples by mechanical dissociation, using a plunger to push tissues through a cell strainer (70-µm nylon mesh). Spleen, lymph node and when necessary, tumor samples were treated with eBioscience 1X RBC Lysis Buffer (Thermo Fisher Scientific, Waltham, MA). An aliquot of each tumor single-cell suspension was taken for cell count. Cells were stained for 20 min with BD Horizon™ BUV395 Rat Anti-Mouse CD45 antibody (BD Biosciences, San Jose, CA; Cat. No. 564279). Cells were resuspended in BD Pharmingen™ Stain Buffer (FBS) containing 1 μg/ml Propidium Iodide (Thermo Fisher Scientific; Cat. No. P3566) for dead cell exclusion and mixed at a 1:1 (v/v) ratio with 123count eBeads™ Counting Beads (Thermo Fisher Scientific; Cat. No. 01-1234-42), before measurement by flow cytometry (see below).

**Figure 1:**
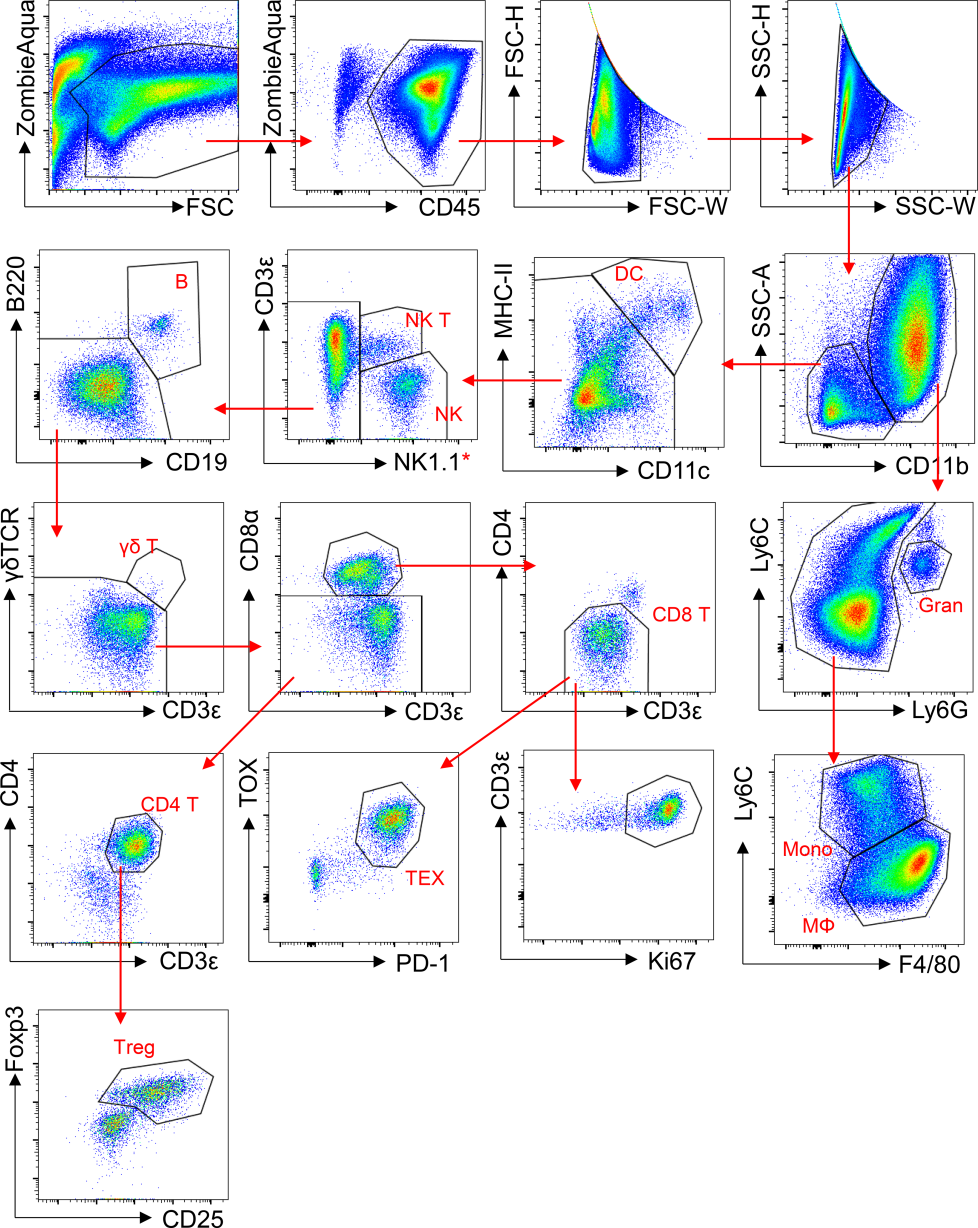
Gating strategy for flow cytometry analysis of tumor-infiltrating lymphocytes (TILs). Single-cell suspensions were prepared from tumors, and cells were stained with fluorochrome-conjugated antibodies against surface and intracellular markers as indicated. Data were acquired on a BD LSRFortessa X-20 flow cytometer and plotted using FlowJo 10 software. Red arrows indicate sub-gating. DC, dendritic cell; NK T, Natural Killer T cell; NK, Natural Killer cell; B, B cell; γδ T, gamma-delta T cell; CD8 T, CD8^+^ T cell; CD4 T, CD4 T cell; TEX, Exhausted CD8^+^ T cell; Treg, regulatory T cell; Gran, Granulocyte; Mono, Monocyte; MΦ, Macrophage.

For dead cell exclusion, the resultant single-cell suspensions were treated with Zombie Aqua Fixable Viability kit (Biolegend, San Diego, CA) according to manufacturer’s instructions. Fc receptors were blocked using the TruStain FcX antibody (Biolegend, San Diego, CA) before staining for 20 min at 4-8 °C in the dark with fluorochrome-conjugated antibodies (**Supplementary Table 1**). Following staining, cells were fixed using FluoroFix™ Buffer (Biolegend, San Diego, CA). To stain for FoxP3 (Forkhead Box P3) and Ki67, intracellular staining was performed using the eBioscience™ Foxp3 Transcription Factor Staining Buffer Set (Thermo Fisher Scientific, Waltham, MA) following manufacturer’s instructions. The stained cells were analyzed using the BD LSRFortessa™ flow cytometer (BD Biosciences, San Jose, CA). Data was analyzed using FlowJo 10 (FlowJo, LLC, Ashland, OR). The gating strategy for the flow cytometric analysis is presented in **Figure 1** (tumor), **Supplementary Figure 1A** (spleen), and **Supplementary Figure 1B** (tumor-draining lymph nodes, TDLN).

### 2.7 scRNA-seq experiments

Tumors from treated mouse models were harvested on day 6 after RT and dissociated as described for flow cytometry (above) to obtain single-cell suspensions. Immune cells were isolated from cell suspensions using CD45 (TIL) MicroBeads, mouse (Miltenyi Biotec, San Diego, CA; Cat. No. 130-110-618) following manufacturer’s instructions. Isolated cells were counted using Cellaca MX Cell Counter (Nexcelom Bioscience, Lawrence, MA). Cells were processed for scRNAseq using the 10x Genomics chromium v3 3’ chemistry pipeline according to the manufacturer’s instructions. Cells were resuspended in 0.04% BSA in PBS at a concentration of 1 × 10^6^ cells/ml. Cells were loaded onto a Chromium Single Cell B Chip (10x Genomics, Pleasanton, CA) to aim for target cell recovery of 8 × 10³ cells. Library construction was performed according to Chromium Single Cell 3' GEM, Library & Gel Bead Kit v3 protocol (10x Genomics). Libraries were sequenced using the NovoSeq 6000 platform (Illumina; San Diego, CA) aiming for a minimum of 200 × 10^6^ reads per library (25,000 reads per cell).

#### 2.7.1 scRNA-seq analysis

Raw sequencing reads were processed into count data using the Cell Ranger v4 (10X Genomics, Pleasanton, CA) and then further analyzed using Seurat 3 (31) with its default setting for data normalization and filtering. To align cells across different samples from the same model, Canonical correlation analysis (CCA (31)) was performed using the top 20 CCA components. Then cell clusters were identified based on recommended resolution in Seurat 3 on the aligned CCA space. Top signature genes in each cell cluster were evaluated to assign cell cluster identity during a manual review. Visualization of single-cell RNA-Seq data was performed using Seurat or ggplot2 function in R.

### 2.8 Immunohistochemistry

The animals were euthanized at different time points, and tumors and tissues were harvested and fixed in 10% neutral buffered formalin. Single chromogenic IHC assay for CD8a was performed on 5-micron sections of formalin-fixed and paraffin embedded tumor tissue. Briefly, tissue sections were loaded onto a Leica Bond III instrument and deparaffinized, followed by pretreatment with epitope retrieval solution 2 (Leica Biosystems) for 20 minutes and then blocking buffer for 10 to 20 minutes. The anti-CD8a antibody (rat anti-CD8a, clone 4SM15, 1:2000; catalog number 14-0808-82; Thermo Fisher Scientific, Waltham, MA); or anti-FoxP3 antibody (rat anti-FoxP3, clone FJK-16s, 1:200; catalog number 14-5773-82; Thermo Fisher Scientific, Waltham, MA); was incubated for 20 minutes followed by biotinylated anti-rat linker antibody applied for 15 minutes prior to detection and color visualization by Refine diaminobenzidine polymer. A coverslip was applied on slides and scanned on Aperio AT2 whole-slide scanner (Leica Biosystems, Vista, CA) at 20X magnification.

#### 2.8.1 Whole-slide digital image analysis

All image analysis was performed using Visiopharm Software version 2020.01 (Visiopharm, Hoersholm, Denmark). The viable tumor regions were manually annotated, and a custom analysis protocol package (App) was developed to detect CD8a positive cells. The results were expressed as cell density. For spatial analysis, the X-Y coordinates of all the CD8a positive cells were exported. In addition, the boundary of the viable tumor tissue was also exported.

#### 2.8.2 Biodistribution score

The Biodistribution (BioD) score was calculated as described previously (32). The BioD score measures the relative spatial distribution of the cells of interest with respect to the tumor boundary. The tumor area is split into ten radially symmetric annular zones of equal area and the CD8α cell density in each zone is calculated. Then the annular regions are further binned into three regions namely core, intermediate and periphery and the average CD8α cell density for each region is computed. The BioD score is defined as the pairwise ratios of the average CD8α cell density in the core, intermediate and periphery regions.

### 2.9 Statistical analyses

TGI was assessed in non-clinical wildtype animal model studies using analysis of covariance (ANCOVA). Log-transformed tumor volumes, adjusted for baseline measurements, were compared between radiation therapy (RT) dose groups and the vehicle (0 Gy) control group within each model The null hypothesis stated that RT does not inhibit tumor growth, while the one-sided alternative hypothesis posited that RT results in tumor growth inhibition relative to the vehicle control. In TGI analyses comparing RT effects in the STING-deficient and wild-type MC38-K animal model, ANOVA tests with interaction between treatment and animal model were performed. To preserve growth differences between wildtype and engineered mice no baseline adjustments to the log-transformed tumor volumes were performed. In this analysis, the null hypothesis stated that RT effects are similar between wildtype and genetically modified mice, while the one-sided alternative hypothesis stated that RT tumor growth inhibition is higher in the wildtype animal model. ANOVA/ANCOVA analyses were conducted on the latest study day when all animals remained on study. This approach was taken to minimize potential bias associated with early removal of animals due to welfare concerns.

Time-to-event analyses were conducted to compare RT dose groups with the control group within each animal model. In these analyses, an event was defined as a tumor reaching a volume of 1500 mm³; observations not meeting this criterion were considered censored. Group comparisons were performed using log-rank tests with one-sided p-values to assess differences in time-to-event distributions. Kaplan–Meier curves were generated to summarize and visually represent the time-to-event data for C57BL/6 tumor-bearing mice treated with varying RT doses.

Tumor cell lines treated with varying doses of RT and control were evaluated for *in vitro* confluency. Four-parameter logistic regression models were fitted to replicate confluency curves. The estimated slope parameters from individual replicates were then compared between each treatment group and the control using ANOVA tests. The null hypothesis stated that RT does not inhibit cell growth, while the one-sided alternative hypothesis asserted that RT delays cell growth relative to the control.

Cell population frequencies obtained from flow cytometry assays and BioD scores were analyzed using a two-tiered statistical approach. The Shapiro–Wilk test was first applied to assess the normality of each cell population frequency distribution. When data significantly deviated from normality (p-value < 0.05), a log transformation was applied. If the transformed data met the normality assumption, group comparisons were performed using analysis of variance (ANOVA). If normality was not achieved, non-parametric Mann–Whitney tests were used for pairwise comparisons. The null hypothesis stated that RT does not alter cell population frequencies, while the two-sided alternative hypothesis posited that RT induces changes in cell population frequencies relative to the control. For TIL analysis experiments, tumors were not randomized on the day of treatment to avoid treatment-induced shrinkage that could preclude subsequent TIL isolation. All tumors were ≤5 mm in each dimension to accommodate the collimated irradiation field. Consequently, differences in tumor size between treatment groups were not tested.

Correlation analyses were conducted to evaluate the hypothesis that tumor hot/cold status may predict RT benefit. Spearman correlation coefficients were calculated between TGI estimates and baseline frequencies of CD8^+^ T cell populations or BioD scores, both within individual dose levels and across all dose levels combined. All p-values reported in this manuscript are unadjusted (raw). Statistical significance is denoted as follows: p < 0.05 (*), p < 0.01 (**), and p < 0.001 (***); “ns” indicates non-significant results. No corrections for multiple comparisons were applied in these analyses. The results are intended to support the generation of biological hypotheses which require further validation. We acknowledge the potential for false discoveries and imprecise effect size estimates. All statistical analyses were performed using R software, version 4.5.1. The data is representative of 1–3 experiments. The sample size for each experiment is mentioned in the Figure legend.

## 3 Results

### 3.1 Syngeneic tumor models show differences in immune cell infiltration

To test how immune cell infiltration relates to tumor-targeted RT-induced TGI and survival, we categorized tumors based on overall immune cell, CD8^+^ T cell infiltration and distribution. We chose syngeneic tumor models on two commonly used *Mus musculus* strains, namely BALB/c (CT26 and EMT6, T helper 2-prone) and C57BL/6 (MC38 and B16F10, T helper 1-prone). Because MC38 tumors are not available from ATCC and there may be different strains being used among the scientific community, we included these from two different sources: MC38-R tumors were obtained from Antoni Ribas and MC38-K tumors were obtained from Kerafast.

**Figure 2:**
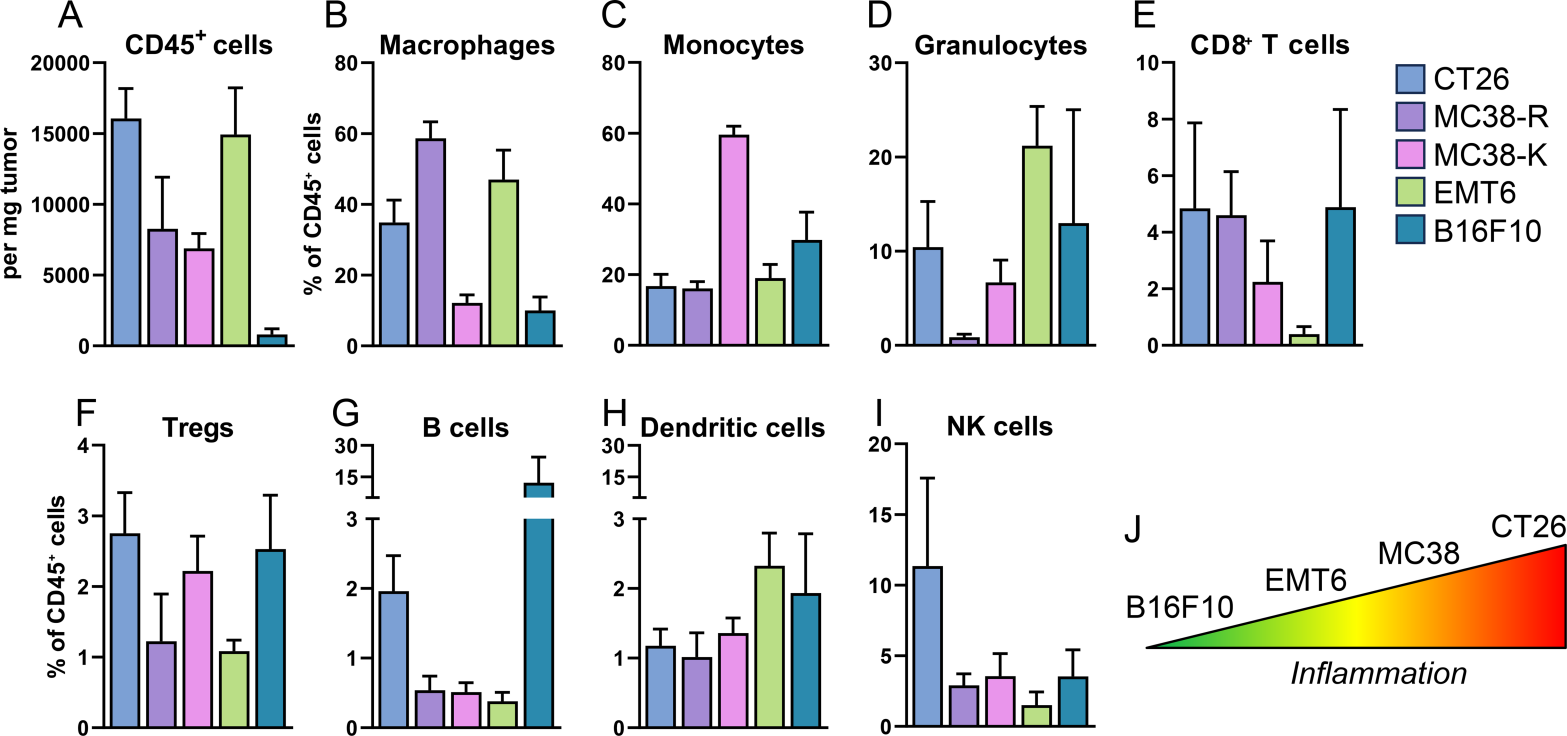
Quantification of immune cell infiltration across syngeneic tumor models (CT26, MC38-R, MC38-K, EMT6 and B16F10). Mice were implanted with cancer cells in the right flank, and tumors were harvested 15–21 days post-implantation. Tumors were processed into single-cell suspensions and analyzed by flow cytometry. **(A)** Absolute number of infiltrating CD45^+^ cells (cells per mg tumor tissue). **(B–I)** Relative frequencies of macrophages, monocytes, granulocytes, CD8^+^ T cells, Tregs, B cells, dendritic cells, and NK cells, respectively, within the CD45^+^ compartment. **(J)** Schematic illustration of inflammation, cold (B16F10, low inflammation) to hot (CT26 being highly inflamed) tumor model. Bars represent mean ± SD (N = 4–6 mice per group).

To compare syngeneic tumor models, we quantified TILs (**Figure 2A**; **Supplementary Figure 2A**) and determined the relative proportions of major immune cell types: macrophages, monocytes, granulocytes, CD8^+^ T cells, regulatory T cells (Tregs), B cells, DCs and NK cells (**Figures 2B–I**, as % of CD45 cells and **Supplementary Figures 2B–I**, as # cells per mg of tumor). We observed strong differences among the five models: B16F10 had the lowest number of immune cell infiltration, whereas EMT6 and CT26 showed the highest infiltration (**Figure 2A**; **Supplementary Figure 2A**). Additionally, the CT26 model had the largest fraction of CD8^+^ T cells (> 4% of CD45 cells; **Figure 2E**). The two MC38 models showed an intermediate immune cell infiltration (**Figures 2A–I**). Strikingly, EMT6 tumors contained the smallest fraction of CD8^+^ T cells (<0.5% of CD45 cells) (**Figure 2E**), despite having a large CD45^+^ immune cell population. Interestingly, B16F10 tumors had >4% of CD8^+^ T cells within the small pool of immune cells in the TME (**Figure 2E**) and was the tumor model with lowest number of CD8^+^ T cells per mg tumor (**Supplementary Figure 2E**). Monocytes and macrophages together accounted for >50% on immune cells present in all tumors (**Figures 2B, C**). **Figure 2J** summarizes inflammatory immune cell distributions across tumor models.

**Figure 3:**
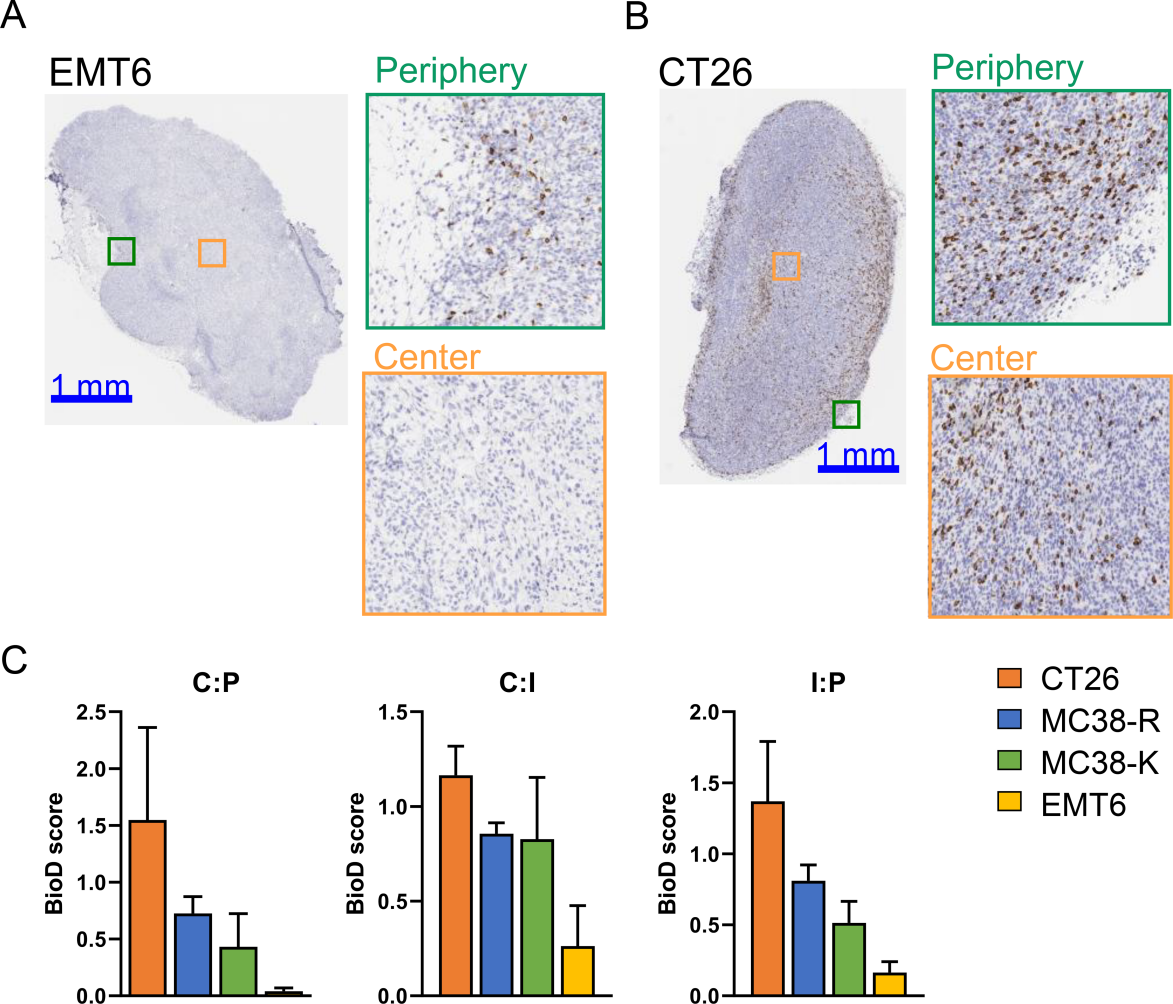
Differential immune cell infiltration across distinct tumor models by immunohistochemistry. Mice were implanted with syngeneic cancer cells in the right flank and tumors were grown for 15 to 21 days before harvesting. **(A)** Representative image of an EMT6 tumor with magnified views of the periphery and the center, showing immunohistochemistry staining for CD8α. **(B)** Similar to **(A)**, but for CT26 tumors. **(C)** Quantification of the BioD scores for the syngeneic tumors models: CT26, MC38-R, MC38-K and EMT6. Bars show average BioD score along with SD (N = 4-6). C, core; I, intermediate; P, periphery of tumor section.

We utilized IHC to assess the intratumoral spatial location of cells expressing CD8α, the vast majority of which represents CD8^+^ T cells. Tumor models showed strong differences in intratumoral distribution, with EMT6 and CT26 tumors representing the extremes: While CD8α^+^ cells were rarely observed towards the center of EMT6 tumors (**Figure 3A**), they were readily observed in CT26 tumors (**Figure 3B**). To quantify this observation, we developed a biodistribution (BioD) score by dividing the tumor area into ten annular zones to compute the average CD8α cell density per zone (**Supplementary Figure 3A**). These zones were then grouped into three regions: core (C), intermediate (I), and periphery (P), with average densities calculated for each. The BioD score was derived from the pairwise ratios of these average densities across the regions (**Supplementary Figure 3B**). Because of the high melanin content of B16F10 melanoma tumors, BioD scores could not be reliably determined (data not shown). For all pairwise comparisons (C:P; C:I; I:P), CT26 showed a BioD score of >1, indicating an enrichment of CD8α^+^ cells towards the core of the tumor, relative to the periphery (**Figure 3C**). BioD scores for both MC38 tumor types were >0.5 and <1.0, representing a homogenous CD8α^+^ cell distribution, while EMT6 tumors showed consistently low BioD scores (<0.5), demonstrating an exclusion of CD8α^+^ cells from the tumor center and an enrichment towards the periphery. Based on the characterization of tumors by flow cytometry and IHC, we classified the tumor models from hot to cold phenotype with CT26 representing a hot phenotype and B16F10 a cold phenotype (**Figure 2J**).

### 3.2 Immunologically hot tumors show stronger TGI after RT than cold tumors

**Figure 4:**
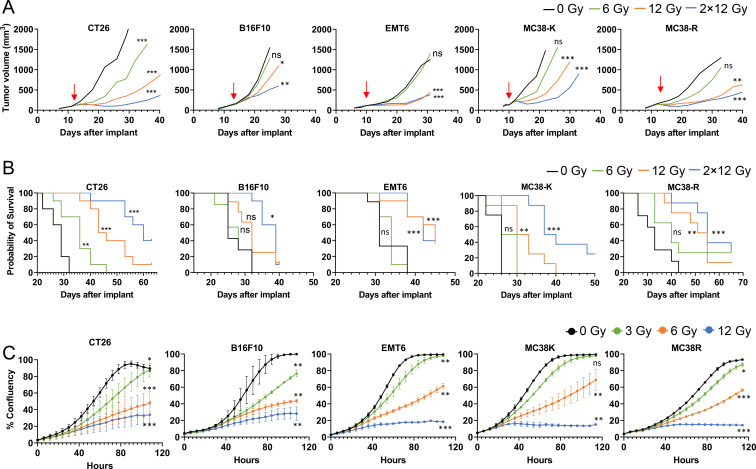
Targeted radiation therapy (RT) leads to dose dependent tumor growth inhibition (TGI) and survival. **(A)** Growth of the indicated syngeneic tumor models *in vivo* treated with the indicated doses (6 Gy - Low; 12 Gy – Intermediate; 2 X 12 Gy – High) of RT. Arrow indicates day of first dose. A one-tailed ANCOVA, adjusting for baseline tumor volume, was employed to test tumor growth inhibition versus control (0 Gy group) on the last day all mice within the same mouse model study were alive. Asterisks denote statistical significance: *p < 0.05, **p < 0.01, and ***p < 0.001; N = 8-10/group. **(B)** Kaplan-Meier curves summarizing time-to-event analyses of C57BL/6 tumor-bearing mice treated with different doses of RT. Events were defined as tumors reaching 1500 mm^3^, otherwise observations were treated as censored. Log-Rank tests were employed to compare survival data time-to-event curves of treatments versus control (0 Gy). Asterisks denote statistical significance: *p < 0.05, **p < 0.01, and ***p < 0.001; N = 8-10/group. **(C)** Tumor cell lines were treated with the indicated dose (3 Gy – Very Low; 6 Gy - Low; 12 Gy – Intermediate) of RT and observed for confluency. Mean of two individual experiments with ± SEM is shown (N = 2). The slope parameter estimates from a 4-parameter logistic regression fit were compared between individual treatment versus control (0 Gy) groups using a t-test. Asterisks denote statistical significance: *p < 0.05, **p < 0.01, and ***p < 0.001 and ns, not significant.

Mice with established tumors (B16F10, CT26, EMT6, MC38-R and MC38-K) were treated with low (6 Gy), intermediate (12 Gy) and high (2 × 12 Gy, 24 Gy given in two fractions on two consecutive days) dose of tumor-targeted RT and evaluated for *in vivo* TGI (**Figure 4A**; **Supplementary Table 2**: TGI) and survival benefit (**Figure 4B**). Compared with untreated tumors, RT led to a dose-dependent TGI (**Figure 4A**) and a significant increase in survival (**Figure 4B**) for CT26 (hot) tumors at all doses evaluated, with TGI values of 64.8%, 83.9%, and 91.1% for low, intermediate, and high-dose RT, respectively. In the other tumor types, only intermediate- and high-dose RT led to significant TGI and survival benefit. The weakest RT response was seen in B16F10 tumors (cold), with TGI values of -0.4%, 47.1%, and 61.3% for low, intermediate, and high-dose RT, respectively; only high-dose RT led to a significant increase in survival. EMT6, as well as both MC38 tumors both responded strongly to intermediate and high-dose RT, showing similar TGI levels at both doses (EMT6: 86.6% vs. 83.0%; MC38-R: 72.9% vs. 84.1%; MC38-K: 76.7% vs. 86.4%), suggesting intermediate dose was sufficient to reach maximal benefit.

To understand the growth inhibitory effects of radiation on the cancer cells per se., in the absence of immune control, we treated monolayer cells *in vitro* with very low (3 Gy), low (6 Gy) and intermediate (12 Gy) irradiation (**Figure 4C**), using lower doses than *in vivo*, because cultured cells are more radiosensitive. Interestingly, B16F10 cells were most strongly affected by irradiation, a response comparable to CT26 cells. EMT6, MC38-K, and MC38-R cells tolerated low-dose irradiation but were sensitive to intermediate-dose treatment. For all cell lines, high-dose irradiation almost completely blocked tumor cell growth *in vitro*.

In summary, we found that intrinsic cell sensitivity to irradiation is not an accurate predictor for *in vivo* TGI by RT. After normalizing for dose, *in vivo* TGI demonstrated a reproducible positive association with CD8^+^ T-cell frequency and BioD scores (**Supplementary Table 3**). A striking example is provided by cell lines B16F10 and CT26, which exhibit similar sensitivity to *in vitro* irradiation. However, when observing *in vivo* tumor growth, tumors derived from cell line B16F10 display relatively lower sensitivity to RT compared to CT26-derived tumors suggesting that the TME and TILs could be contributing to *in vivo* TGI.

### 3.3 Tumor targeted RT induces pro- and anti-inflammatory changes in the tumor immune microenvironment

**Figure 5:**
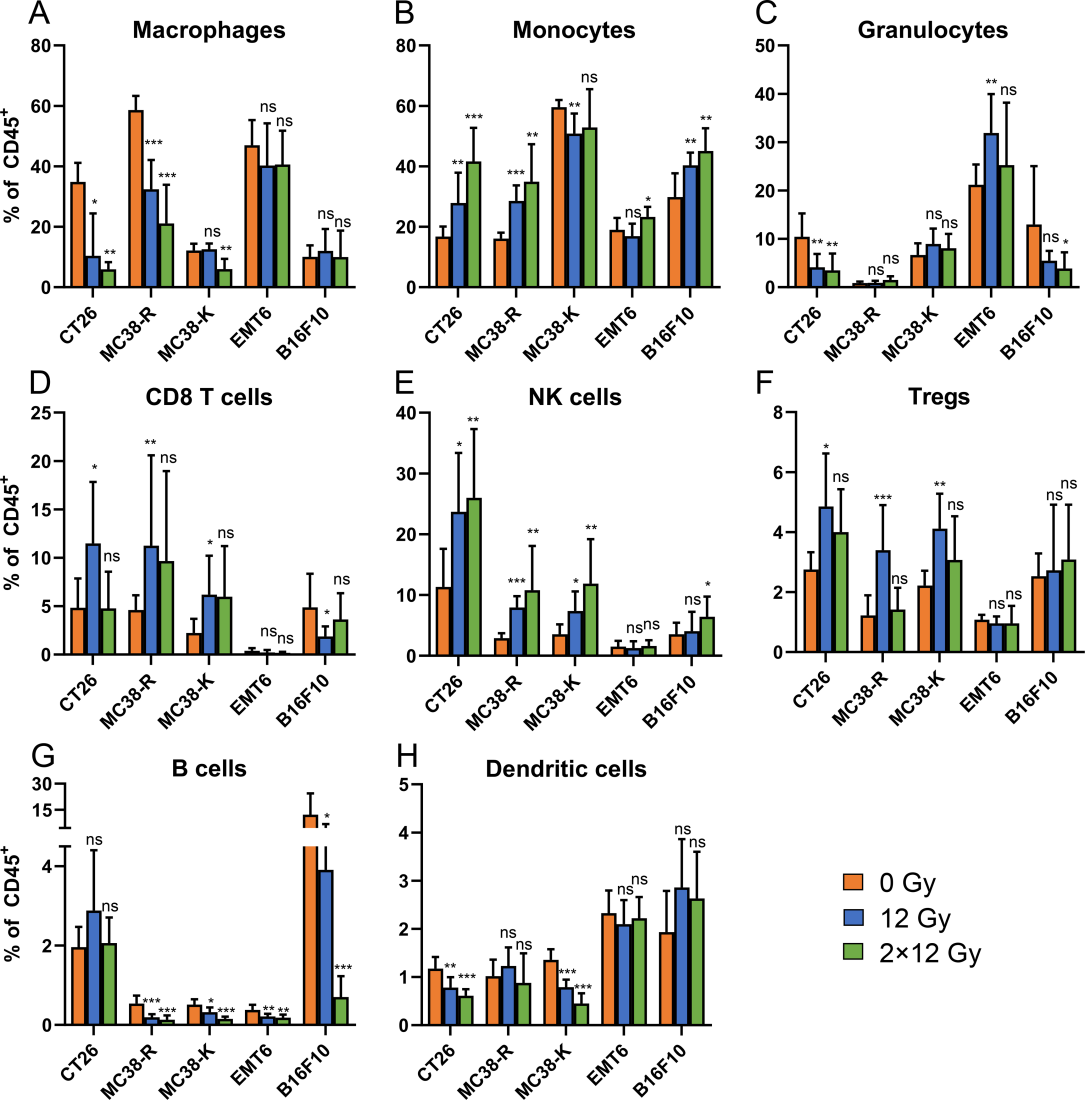
Targeted RT leads to substantial changes in the tumor immune microenvironment. Mice were implanted with syngeneic cancer cells and treated with the indicated dose of RT. Six days after RT, TILs were analyzed by flow cytometry. **(A)** Macrophages, **(B)** Monocytes, **(C)** Granulocytes, **(D)** CD8^+^ T cells, **(E)** NK cells, **(F)** Treg cells, **(G)** B cells and **(H)** DCs. Statistical analysis was done for every cell population relative to control (0 Gy). For all cell types, Shapiro-Wilk tests were used to pre-test the assumption of normal data distribution. Departures from normality (Shapiro-Wilk p-value < 0.05) were alleviated by a log transformation applied to the data. Whenever the normality assumption was met (original or log-transformed data), pairwise comparisons across mouse models were then performed using t-tests. If the normality assumption was still not met after transformation, a rank-based test (Mann-Whitney) was used for the pairwise comparisons. Asterisks denote significance: *p < 0.05, **p < 0.01, ***p < 0.001 and ns, not significant; ± SD is shown, N = 6–8 samples/group.

To better understand immune response dynamics and therapeutic implications, we analyzed TILs six days after intermediate and high-dose RT using multiparametric flow cytometry. Weights of tumors analyzed are shown in **Supplementary Figure 4A**. Treatment with RT induced complex tumor model-specific changes in immune cell infiltration (**Figures 5A–H**). Analysis of the relative contribution of each immune cell type (macrophages, monocytes, granulocytes, CD8^+^ T cells, NK cells, Tregs, B cells and DCs) to this immune cell infiltration by multi-parametric flow cytometry revealed a strong diversity between tumor types.

Macrophages and monocytes, which often adopt an immunosuppressive phenotype in tumors (33), were prominent in CT26, MC38, and EMT6 tumors. RT strongly reduced macrophages in CT26 and MC38-R tumors but not in EMT6 tumors (**Figure 5A**). Macrophages were much less frequent in MC38-K and B16F10 tumors, and only high-dose RT reduced their abundance in MC38-K tumors.

Monocytes, precursors to macrophages (34) and M-MDSCs, contribute to an immunosuppressive TME. Neutrophils, a type of granulocyte, can also acquire immunosuppressive function as PMN-MDSCs (35). Distinguishing MDSCs from neutrophils or monocytes by flow cytometry remains challenging due to substantial phenotypic overlap (36). Canonical surface markers are broadly expressed across mature myeloid cells and hematopoietic precursors. High-dimensional profiling revealed that cells within conventional MDSC gates exhibit extensive heterogeneity and lack a distinct transcriptional or functional signature (37). MC38-K tumors had a large monocyte fraction, which decreased after intermediate-dose RT (**Figure 5B**). Monocytes increased in CT26, MC38-R, and B16F10 after both RT doses, while in EMT6, only high-dose RT caused an increase. Granulocytes, abundant in EMT6 tumors, increased slightly after intermediate-dose RT (**Figure 5C**). In CT26, both intermediate- and high-dose RT led to their reduction. In B16F10, only high-dose RT caused a significant reduction, with intermediate-dose RT showing a similar trend.

CD8^+^ T cells are the key effector cells and play an important role for the abscopal effect (38). These were increased following intermediate-dose RT in CT26, MC38-R and MC38-K, but decreased in B16F10 tumors (**Figure 5D**; **Supplementary Table 2**: CD8T). No changes were observed in EMT6 tumors. Surprisingly, high-dose induced no changes in CD8^+^ T cells in any tumor type. The proportion of exhausted CD8^+^ T (T_EX_) cells (PD-1^+^TOX^+^) did not increase following RT (**Supplementary Figure 4B**). NK cells, highly cytotoxic effector cells but often inhibited by an immunosuppressive TME (39), increased in CT26 and MC38 after RT (**Figure 5E**; **Supplementary Table 2**: NK). Only high-dose RT increased NK cells in B16F10, with no change in EMT6. No tumor type showed a reduction in NK cells post-RT.

Strikingly, FoxP3^+^ regulatory T (Treg) cells, CD4 T cells that suppress anti-tumor immune responses, were not reduced in any tumors following radiation. Instead, radiation increased the fraction of Tregs among immune cell infiltrates in CT26, MC38-R and MC38-K tumors (**Figure 5F**).

IHC analysis of the intermediate–dose RT cohort confirmed the flow cytometry findings: CD8α staining was sparse in B16F10 and EMT6 tumors, compared with MC38 and CT26 tumors. IHC of tumors 6 days after RT showed an increased frequency of CD8α^+^ cells (**Supplementary Figure 5A**). Similarly, FoxP3^+^ cells, which were less frequently observed than CD8α^+^ cells, were increased after RT (**Supplementary Figure 5B**).

Intra-tumoral B cells, which are commonly immunosuppressive were abundant in B16F10 tumors, whereas they were a smaller fraction of immune cells in MC38-R, MC38-K, and EMT6 tumors. There was a dose-dependent reduction in B cells following RT in all tumor types except for CT26 (**Figure 5G**). DCs were reduced after RT in CT26 and MC38-K tumors with either dose, but unchanged in the other tumor models (**Figure 5H**). In summary, RT induced complex and, in some cases, inverted dose-response effect and tumor-specific alterations in immune cell infiltration. Macrophages and DCs were reduced in select tumors, whereas monocytes and granulocytes increased in others, and key effector cells such as CD8^+^ T cells, NK cells, and Tregs were modulated in a dose- and context-dependent manner.

### 3.4 Tumor-target RT leads to systemic immunological changes

Changes in the cellular composition of distal immunological tissues indicate systemic effects of tumor-targeted RT. We therefore analyzed spleens of tumor bearing mice after treatment. RT induced distinct, but modest immune changes in the spleen, with the MC38 tumors showing the most pronounced effects (**Supplementary Figures 6A–J**). For example, NK cells increased significantly in MC38-R mice with both RT doses (**Supplementary Figure 6D**).

We also analyzed the TDLN (**Supplementary Figures 7A–G**). Lymph nodes of young healthy mice are dominated by two populations: 65 – 75% T cells and 10 – 15% B cells (40, 41). In the TDLNs of mice with MC38 tumors, there were notably fewer T cells at baseline. RT induced only marginal changes in T and NK cell populations in TDLNs (**Supplementary Figures 7A, F**). When analyzing overall B cells, we noted only a slight reduction after intermediate-dose RT in MC38-R and EMT6 tumors. Upon activation by their cognate antigen, B cells can initiate the formation of germinal centers (GCs), which are structures in secondary lymphoid organs where GC B cells proliferate and undergo selection to produce high-affinity antibodies (41). In tumors, GCs form within tertiary lymphoid structures, but since syngeneic tumor models usually lack these structures, we analyzed GC B cells in TDLNs instead (42). RT induced pronounced alterations in GC B cells (**Supplementary Figure 7E**). In mice bearing CT26 or EMT6 tumors, both doses prompted a decline in GC B cells, with high-dose RT resulting in a more marked reduction for EMT6 TDLNs. Although TDLNs of B16F10 tumors initially contained very few GC B cells, intermediate-dose RT further diminished this population, while high-dose RT exhibited no discernible effect. Intriguingly, intermediate-dose RT significantly increased GC B cells in mice bearing both types of MC38 tumors. Conversely, high-dose RT did not enhance, and notably diminished, this population in TDLN of MC38-R tumors.

### 3.5 RT leads to an increase in intratumorally proliferating CD8^+^ T cells

**Figure 6:**
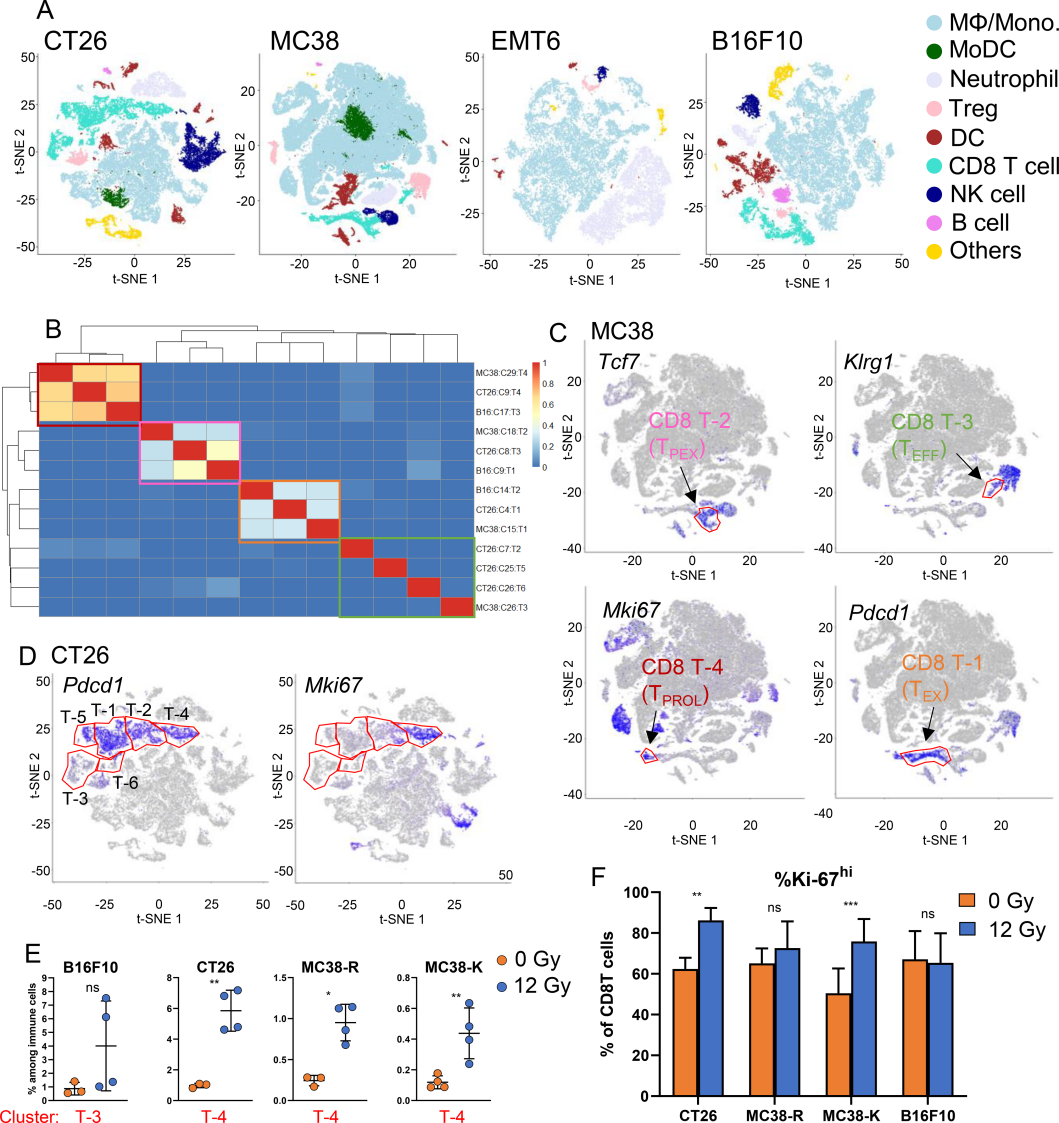
Targeted RT leads to an intratumoral increase in proliferating T cells. **(A)** Overview of immune cell populations as t-distributed stochastic neighbor embedding (t-SNE) plot in indicated syngeneic tumor models. For graphs **(A–C)**, the datasets from the MC38 variants -R and -K was combined for joint clustering to enable identification of shared transcriptional populations and is represented as MC38 in the graphs. **(B)** Hierarchical clustering of CD8^+^ T cell populations as identified by single cell RNA (scRNA) sequencing. Four major clusters are highlighted with different colored boxes in the heatmap. **(C)** Representative t-SNE plots of 4 T cell clusters in MC38 tumors. *Tcf7* – a marker of T_PEX_ cells; *Klrg1* – a marker of T_EFF_ cell; *Mki67* – a marker of proliferating T cell; *Pdcd1* – a marker of T_EX_ cells. **(D)** CD8^+^ T cell clusters from CT26 tumor model expressing proliferation-associated and exhaustion-associated genes in control or RT-treated tumors. **(E)** Impact of RT on fractions of respective T cell clusters in B16F10, CT26, MC38-R and MC38-K tumor models. **(F)** Effect of RT on high *Ki-67* expressing CD8^+^ T cells in the indicated tumor types as measured by flow cytometry. Shapiro-Wilk tests were used to pre-test the assumption of data normal distribution. Whenever the normality assumption was met (Shapiro-Wilk p-value ≥ 0.05), pairwise comparisons across mouse models were then performed using t-tests. If the normality assumption was still not met, a rank-based test (Mann-Whitney) was used for the pairwise comparisons. Asterisks denote significance: *p < 0.05, **p < 0.01, ***p < 0.001, and ns, not significant; ± SD is shown, N = 3-4/group.

Flow cytometric analysis revealed an increase in CD8^+^ T cells in the TME after intermediate-dose (12 Gy) RT across multiple tumor types (**Figure 5D**). Therefore, we chose intermediate-dose RT to compare and characterize the TILs from CT26, MC38-R, -K, EMT6, and B16F10) tumors. We isolated CD45^+^ TILs from syngeneic tumors 6 days after RT and analyzed their gene expression using single-cell RNA sequencing (scRNA-seq). We combined the datasets from the MC38 variants R and K for joint clustering (labeled as MC38) to identify shared transcriptional populations in a common space, while datasets from the other tumor models were clustered independently to optimize resolution of model-specific cell states. To visualize immune cell diversity across the tumor models we used t-distributed stochastic neighbor embedding (t-SNE) plots and differentially expressed genes for each cluster can be found in **Supplementary Table 4**. Most TIL populations were found in all tumors, with the exceptions of B cells and CD8^+^ T cells, which were absent from the sampled cells isolated from EMT6 tumors (**Figure 6A**). Their absence from the t-SNE map likely reflects their low abundance, causing them to cluster with transcriptionally similar populations rather than forming distinct groups. Consistent with flow cytometry, monocytes were the predominant population of cells by scRNA-seq.

To identify similar CD8^+^ T cell clusters across different tumor types, we performed hierarchical clustering and identified 4 superclusters with similar gene expression (**Figure 6B**). To illustrate the identity of the superclusters, we used MC38 TILs as an example and examined the expression of established marker genes (**Figure 6C**). Among the identified clusters, CT26-T2, CT26-T5, CT26-T6, and MC38-T3 (**Figure 6C**) expressed *Klrg1*, a marker gene associated with effector T cells (T_EFF_). These cells are highly functional and induce tumor cell death through the secretion of cytotoxic molecules and cytokines (43, 44). In contrast, no T_EFF_ clusters were detected in EMT6 and B16F10 tumors among the cells sampled, consistent with their overall low CD8^+^ T cell infiltration.

Progenitor or precursor exhausted T cells (T_PEX_), identified by the transcription factor *Tcf7*, constitute a self-renewing subset that gives rise to exhausted T cells (T_EX_) upon stimulation (45–49). Clusters MC38-T2 (**Figure 6C**), CT26-T3, and B16-T1 contained T_PEX_ cells; however, no such cluster was detected among cells sampled from EMT6 tumors.

Clusters MC38-T4 (**Figure 6C**), CT26-T4, and B16-T3 expressed marker genes associated with proliferation. Proliferating T cells (T_PROL_), characterized by *Mki67*, are actively dividing and represent a transitional phase between precursor and T_EFF_ or T_EX_ cells (50, 51). Chronic antigen stimulation in infections and cancer leads to T cell exhaustion, a process regulated by the transcription factor TOX (52–54). T_EX_ cells, marked by high *Pdcd1* expression, progressively lose their effector functions and proliferative capacity. Notably, clusters MC38-T1, CT26-T1, and B16-T2 exhibited marker gene signatures of T_EX_ cells.

CT26 tumors contained the most CD8^+^ T cell clusters, three of which showed low similarity to other clusters. CT26-T4 expressed both *Mki67* (a proliferation marker) and Pdcd1 (a marker of T_EX_ cells) (**Figure 6D**). CT26-T2 partially expressed *Mki67*, while CT26-T5 expressed high *Pdcd1*. CT26-T6 expressed neither *Mki67* nor *Pdcd1* (**Figure 6D**).

We noted that RT induced a strong increase in the fraction of clusters with proliferative gene signatures, except for B16F10, where only 2 of 4 tumors analyzed showed this change (**Figure 6E**; **Supplementary Table 2**: T_PROL_). Although Ki-67 staining is widely used to assess proliferation, and its half-life in cancer cells has been reported to be short (1–1.5 hours), studies in T cells have shown that Ki-67 protein can remain stably expressed after cell division (55, 56). Consistent with this, we observed substantially higher Ki-67 expression by intracellular flow cytometry (**Figure 6F**). Notably, RT led to an increase in Ki-67^+^ CD8^+^ T cells in tumor CT26 and MC38-K, but not in MC38-R and B16F10, although a trend was observed in MC38-R.

Across the remaining T cell clusters, treatment was associated with a significant decrease in T_PEX_ cells in CT26 tumors (**Supplementary Figure 8A**), a significant increase in T_EX_ cells in MC38R tumors (**Supplementary Figure 8B**), and a significant increase in T_EFF_ cells in MC38R as well as in one effector T cell subset in CT26 tumors (**Supplementary Figure 8C**).

Therefore, despite a much different expression baseline expression level, we found that flow cytometry confirms the scRNA-seq finding that RT leads to an increase in (recently) dividing CD8^+^ T cells.

### 3.6 Proliferating macrophages are reduced following RT

**Figure 7:**
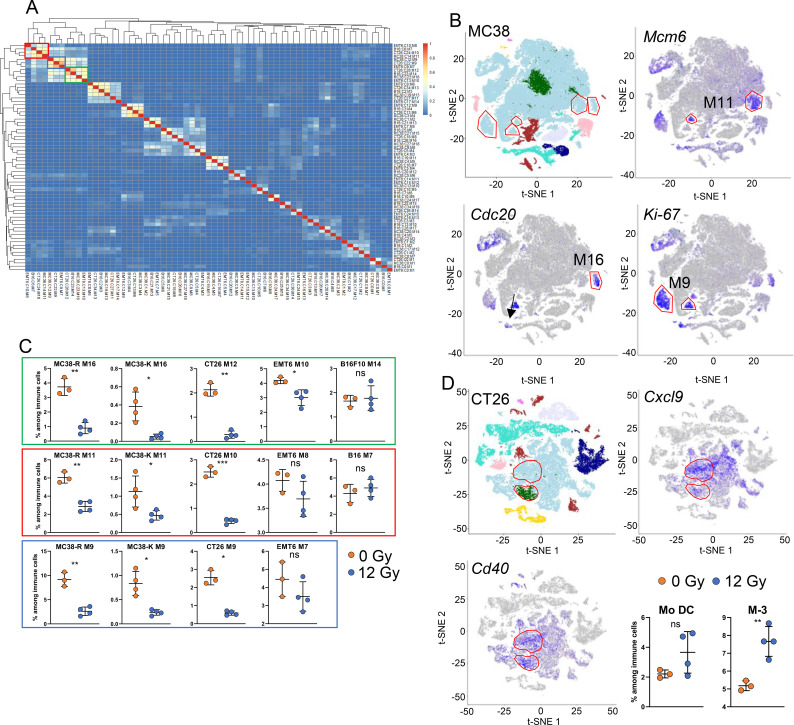
Targeted RT impacts intratumoral proliferating macrophages. **(A)** Hierarchical clustering of macrophage and monocyte populations as identified by scRNA sequencing. The three super clusters with high proliferation gene signature are highlighted in colored boxes in left top corner. For graphs A-B, the datasets from the MC38 variants -R and -K were combined for joint clustering to enable identification of shared transcriptional populations and is represented as MC38 in the graphs. **(B)** Expression of representative genes (*Mcm6, Cdc20* and *Ki67*) defining three proliferating macrophage populations and their clusters in MC38 tumors. **(C)** Effect of RT on the proliferating macrophage and monocyte populations in respective clusters across tumor models. **(D)** Pro-inflammatory myeloid populations (*Cxcl9^+^* and *Cd40^+^* cells) in CT26 tumor model: Clusters marked represent the MoDCs and M-3 macrophages in CT26 model. Graphs show MoDCs and M-3 macrophage fractions in control or RT treated CT26 tumors. For **(C)** and **(D)**, pairwise comparisons 12 Gy vs 0 Gy across cell populations and mouse models were performed using t-tests. Asterisks denote significance: *p < 0.05, **p < 0.01, ***p < 0.001, and ns, not significant; ± SD is shown, N = 3-4/group.

Hierarchical clustering of macrophage clusters from all tumor models resulted in a total of 65 clusters (**Figure 7A**), showcasing the complexity of the monocyte derived immune cells. We found three super clusters containing monocyte/macrophage populations with high expression of signature genes of proliferation (**Figure 7A**, top left corner of the heatmap). Similar to what was found for CD8^+^ T cells, these showed the highest inter-tumoral similarity characterized by hallmark genes of proliferation and could be categorized into three distinct groups. The t-SNE plot and clusters of MC38 model as an example is shown in **Figure 7B**. The first group of macrophage clusters, denoted by green box (**Figure 7A**), exhibited pronounced upregulation of *Cdc20* and *Birc5*. The second cluster, marked by the blue box, exhibited heightened expression of *Mki67* and *Top2a*. Lastly, the third cluster, indicated by the red box, displayed the highest expression levels of minichromosome maintenance genes, such as *Mcm6* and *Mcm5*. Most of these populations were strongly reduced after RT (**Figure 7C**, boxed with matching colors with clusters depicted in **Figure 7A**; **Supplementary Table 2**: MPROLg, MPROLr and MPROLb). However, no reduction was observed for the two B16F10 populations (B16-M14 and B16-M7) belonging to the three proliferating macrophage groups.

In case of EMT6, a reduction was observed only for EMT6-M10, but not for the other two populations EMT6-M8 and EMT6-M7 (**Figure 7C**). Since CT26 tumors showed particularly strong T cell infiltration and TGI after RT, we were interested in pro-inflammatory myeloid populations (*Cxcl9*^+^ and *Cd40*^+^ cells) in these tumors (**Figure 7D**). In two of the four tumors analyzed monocytic dendritic cells (moDCs) increased. Strikingly, macrophage population M3, which is characterized by high expression of *Cd40* and *Cxcl9*, has recently been shown to be a key population for the response to CPI by recruitment of protective CXCR3^+^ T cells (57). Among the diverse immune cell types, myeloid and CD8^+^ T cells and their subsets were significantly altered due to RT. Thus, we evaluated the STING and HPK1 pathways which are key pathways for myeloid/macrophage and CD8^+^ T cell function and activity.

### 3.7 STING deficiency affects TGI in tumors containing large baseline population of myeloid cells/macrophages with interferon response signature

**Figure 8:**
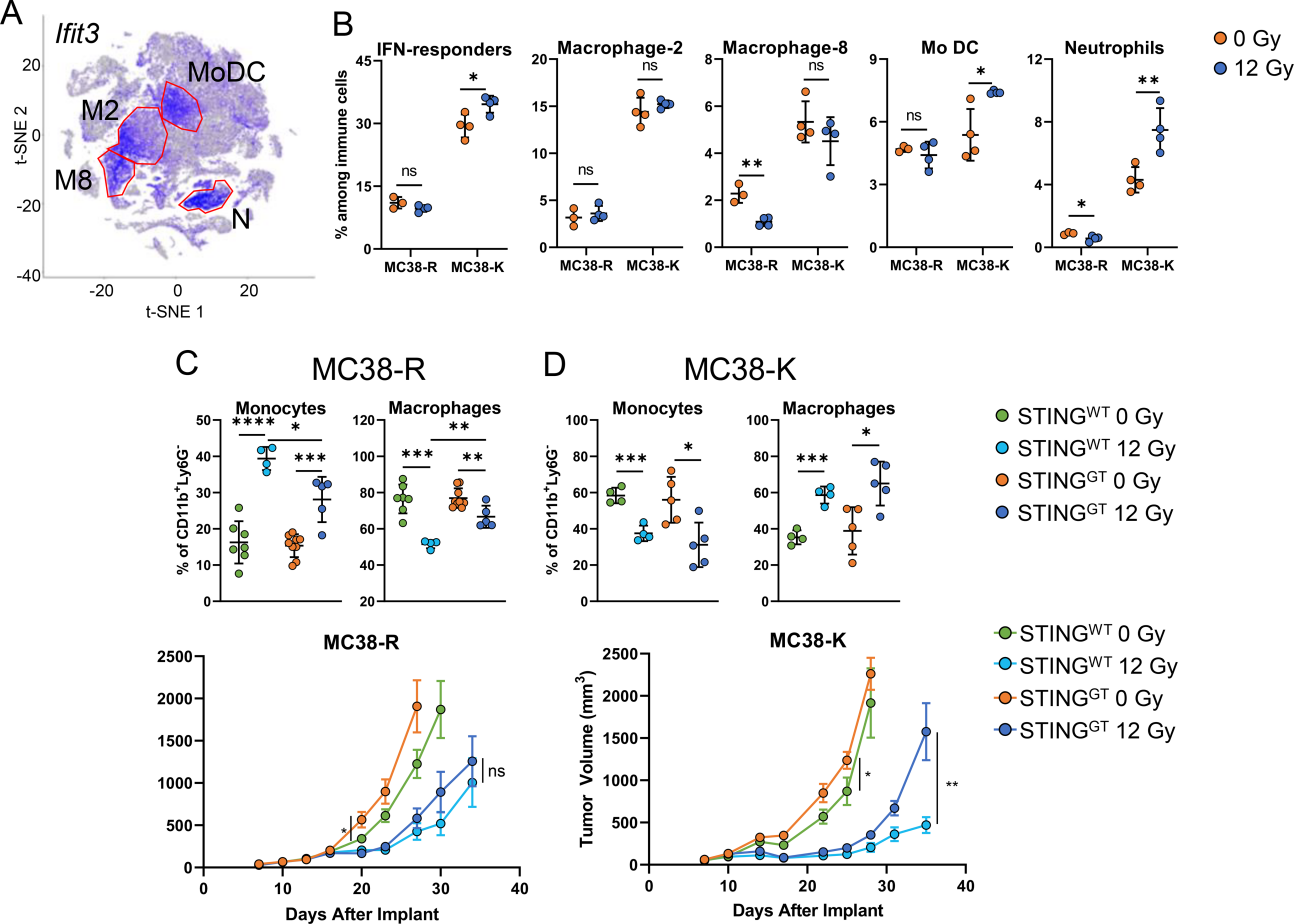
Role of interferon-response gene expressing myelocytes on TGI after RT in MC38-R and MC38-K tumors. **(A)** Expression of *Ifit3* in MC38-R and –K tumor infiltrating leukocytes, four populations (moDC, neutrophils, M2 and M8 clusters) with high *Ifit3* expression are highlighted. These populations were identified by clustering in Seurat 3 using the recommended resolution on the aligned CCA space (as described in Methods). **(B)** Effect of RT on the IFN-responders and the four myeloid cell populations with high expression of type-I IFN genes in MC38-R and MC38-K tumors. Statistical significance for comparisons between RT-treated and control (0 Gy) groups is indicated as: ns = not significant, *p < 0.05, **p < 0.01, ***p < 0.001; ± SD is shown, N = 3-4/group. **(C)** Effect of RT on monocytes and macrophages and tumor growth inhibition in wild-type and STING-GT mice implanted with MC38-R tumors. **(D)** Effect of RT on monocytes and macrophages and tumor growth inhibition in wild-type and STING-GT mice implanted with MC38-K tumors. For **(C)** and **(D)**, pairwise comparisons of treatment versus control (0 Gy) across cell populations and mouse models were performed using t-tests. Asterisks denote significance: *p < 0.05, **p < 0.01, ***p < 0.001, and ****p < 0.0001; ± SD is shown, N = 4–6/group. For tumor growth inhibition graphs, one-tailed ANOVA tests, were employed to test tumor growth inhibition versus control (0 Gy group) on the last day all mice within the same mouse model study were alive. ns = not significant; Asterisks denote statistical significance: *p < 0.05, and **p < 0.01; ± SEM is shown, N = 8–10/group.

We identified four clusters (macrophage/monocyte clusters 2 and 8, as well as neutrophils and moDCs) in the MC38 tumors that we define as “IFN-responders” based on higher expression of IFN-response genes (*Ifit3*) compared to all other immune cell clusters (**Figure 8A**). We noticed a strong difference (~2×) in the cluster sizes between MC38-R and MC38-K TILs, with MC38-K tumors showing a much larger fraction of these cells than MC38-R tumors (**Figure 8B**). As a whole, these were unchanged in MC38-R tumors following RT but further increased in MC38-K tumors (**Figure 8B**). Macrophage clusters 2 and 8 also expressed high levels of *Ly6c*, consistent with a monocyte phenotype. Following RT treatment, moDCs and neutrophils were significantly increased in MC38-K tumors, while macrophage/monocyte cluster 8 and neutrophil cluster were reduced in MC38-R tumors (**Figure 8B**). Thus, MC38-R and -K provided an interesting opportunity to compare two very similar cell lines and the STING pathway.

Liang et al. found that STING activation recruited MDSCs to the tumor following tumor-targeted RT of MC38 tumors and suppressed the anti-tumor immune response (58). In this study, MDSCs were identified as CD45^+^CD11b^+^Ly6C^+^ cells. Based on the low frequency of Ly6C^+^ cells among CD11b^+^ cells identified by Liang et al., we assumed that these tumors reflect our MC38-R tumors and not MC38-K tumors. To confirm the role of these MDSCs in RT-mediated TGI we utilized STING^GT^ mice. The STING^GT^ mouse carries a point mutation (T596A) in *Sting*, leading to an isoleucine-to-asparagine substitution (I199N) in the STING protein (59).

Interestingly, RT significantly increased monocytes in MC38-R tumors growing in STING^GT^ and wildtype animals, but this increase was significantly reduced in STING^GT^ animals and accompanied by an increased frequency of macrophages compared with wildtype animals (**Figure 8C**). Strikingly, the opposite was observed in MC38-K tumors, where RT decreased Ly6C^+^ monocytes and increased macrophages (**Figure 8D**). Additionally, MC38-R tumors grew faster in STING^GT^ mice than in wild-type mice, but RT-induced TGI was not different between the two strains of mice with MC38-R tumors (**Figure 8C**). Most importantly, MC38-K tumor growth was similar in STING^GT^ and wildtype mice, RT lead to a significantly stronger TGI in STING^WT^ than STING^GT^ mice, showing a synergistic effect (**Figure 8D**). The loss of TGI difference in MC38-R tumors, indicates that recruitment of Ly6C^+^ monocytes diminishes the RT-induced anti-tumor immune response in MC38-R tumors (60), but not MC38-K tumor model.

### 3.8 Combination of HPK1-deficiency with RT leads to synergistic tumor growth control

Since we observed an induction of CD8^+^ T cell expansion following RT, we were interested in testing whether HPK1, a kinase primarily expressed in hematopoietic cells limits RT-induced T cell response. HPK1 limits T cell activity by phosphorylating the adapter protein SLP76 in the TCR pathway (61, 62). Prostaglandin E2 (PGE2), an immunosuppressive molecule found in the TME has been shown to further activate HPK1 (63). Deficiency of HPK1 suppresses tumor growth by elevating the anti-tumor immune response, particularly in combination with anti-PD-L1 therapy (64, 65). Therefore, HPK1 is a promising target for cancer immunotherapy. While HPK1 inhibitors have been previously described, our study aimed to assess the biological consequences of HPK1 ablation. Since small-molecule inhibitors inherently introduce variables such as potency, selectivity, and off-target effects, we opted to use knockout mice to directly assess HPK1 function without confounding factors.

**Figure 9:**
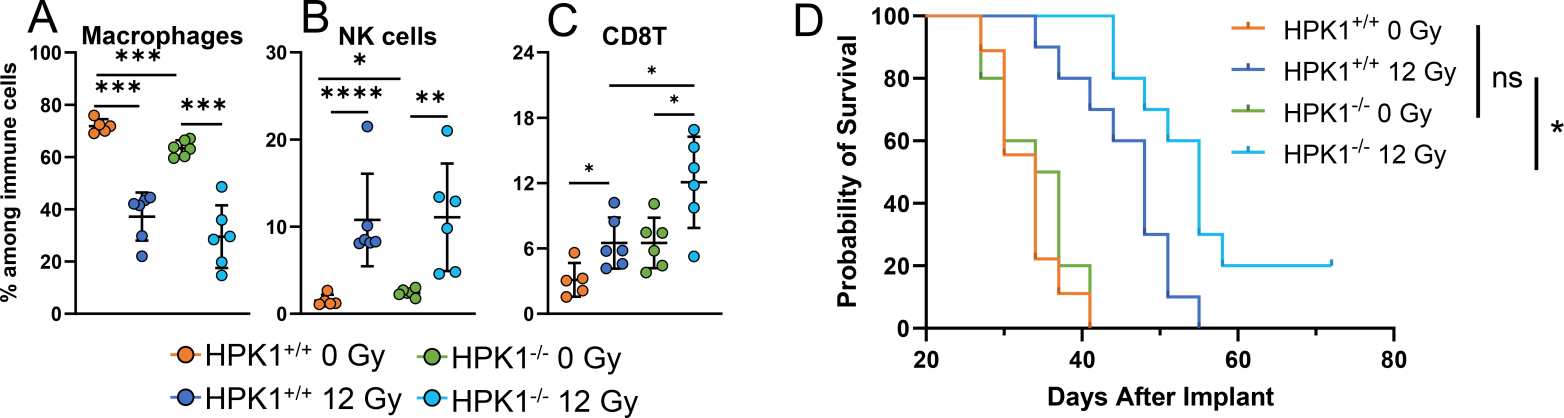
RT leads to strong CD8^+^ T cell tumor-infiltration and increased survival in HPK1-deficient mice. **(A–C)** Indicated immune cell populations in MC38-R tumors of HPK1-deficient or -wildtype mice, six days after RT or control treatment. **(A)** Macrophages, **(B)** NK cells, **(C)** CD8^+^ T-cells. Pairwise comparisons across cell populations were performed using t-tests. Asterisks denote significance: *p < 0.05, **p < 0.01, ***p < 0.001, and ****p < 0.0001; ± SD is shown, N = 4-6/group. **(D)** Kaplan-Meier curves summarizing time-to-event analyses of HPK1-deficient and wildtype mice bearing MC38-R tumors treated with RT or control treatment (n=8 to 10 mice per group). Events were defined as tumors reaching 1500 mm^3^ in volume otherwise observations were treated as censored. Log-Rank tests were employed to compare time-to-event curves of treatments versus control (0 Gy). Asterisks denote statistical significance: *p < 0.05, and ns, not significant; N = 8–10/group.

We tested the effects of RT on TILs in the absence of HPK1 in MC38-R tumors (**Figures 9A–C**). Without RT treatment, MC38-R tumors in HPK1^-/-^ mice contained a slightly reduced fraction of macrophages (**Figure 9A**). In HPK1^+/+^ and HPK1^-/-^ mice, tumor-targeted RT led to a strong and significant reduction of macrophages, but there was no significant difference between treated tumors in both groups. NK cells on the other hand were slightly increased in tumors in HPK1^-/-^ mice, but after RT treatment there was again no difference for this cell population in tumors in HPK1^-/-^ and HPK1^+/+^ animals (**Figure 9B**). Finally, CD8^+^ T cells were significantly increased in tumors growing in HPK1^-/-^ deficient mice (**Figure 9C**). RT led to an increase of the fraction of CD8^+^ T cells in HPK1^+/+^ mice to about the same size as in HPK1^-/-^ mice without treatment, but this population was strongly increased in HPK1^-/-^ after RT, comprising about 12% of the total intra-tumoral immune cell fraction.

While HPK1-deficiency alone did not lead to an increased survival of MC38-R tumor bearing mice, RT synergized with HPK1-deficiency leading to a significantly increased survival (**Figure 9D**). In summary, our data demonstrate that HPK1 deficiency increases CD8^+^ T cell infiltration and synergizes with RT to improve survival, suggesting that the improved outcome may result from an augmented anti-tumor immune response.

## 4 Discussion

Targeted RT has shown great promise not only as a tumor debulking therapy, but also for its potential immunotherapeutic benefit (60). Here, we systematically analyzed the effects of tumor-targeted RT on the anti-tumor immune response in syngeneic murine tumor models. Preclinical murine tumor models show remarkable heterogeneity (66) and are widely used to evaluate novel drug targets (67). Human tumors can be categorized into immune hot, suppressed and cold based on their immune cell infiltration, particularly in relation to CD8^+^ T cell infiltration (68). Other studies investigating the immunological effects of tumor-targeted RT have typically focused on specific cancer indications or specific types of immunotherapy treatments (69). In contrast, our research encompasses immunologically diverse tumor models and examines the effect of RT on them.

Syngeneic mouse tumor models are widely employed in preclinical oncology and provide a diverse panel of immunocompetent, “hot-to-cold” tumors for therapeutic testing (66, 67, 70). Subcutaneous (s.c.) implantation removes the tumor from its native organ, thereby eliminating organ-specific stromal architecture, parenchymal signaling, and resident immune populations (71). Consequently, we did not select models by tissue of origin; instead, we assembled a syngeneic tumor panel based on immunological phenotype. Among the five models examined in this study, CT26 exhibited characteristics of a “hot” and B16F10 a “cold” tumor. Notably, the two MC38 models displayed distinct differences in their myeloid cell populations: the TME of MC38-R contained a large fraction of macrophages, whereas MC38-K tumors contained predominantly monocytes. Additionally, EMT6 tumors exhibited the greatest proportion of granulocytes and very few CD8^+^ T cells.

Flow cytometry cannot assess intra-tumoral localization of immune cells, because this technique requires tumor homogenization. Clinically, IHC is frequently used to determine immune cell infiltration in human tumors (72). Previous studies have demonstrated that in some tumor models, T cells accumulate at the invasive margin, while their presence is notably sparse in the tumor core (66). Similarly, intratumoral immune cell distribution, such as restriction of CD8^+^ T cells to the tumor periphery, is observed in human patients and can predict immunotherapy outcome (68). We quantified CD8^+^ T cell distribution in four tumor types and found that particularly EMT6 tumors exhibited an immune excluded phenotype with CD8^+^ T cells predominantly at the tumor margins, as described previously (73). These findings provide a framework for selecting appropriate tumor models in future studies, depending on whether the focus is on hot or cold tumors, or on specific TIL populations, such as myeloid cell populations.

Interestingly, we did not observe an apparent relationship between *in vitro* radiation resistance and *in vivo* TGI induced by tumor-targeted RT (**Supplementary Table 3**). For example, B16F10 cells were sensitive to RT *in vitro*, whereas B16F10 tumors exhibited significant resistance to RT *in vivo*. Among the tumor types selected for this study, immune cell infiltration at baseline appeared more closely associated with treatment outcome: The hot tumor model CT26 showed strong TGI, strong increase in survival, together with strong increase in CD8^+^ T cell infiltration following RT, whereas the cold tumor model B16F10 showed no CD8^+^ T cell increase following RT, and both TGI and improved survival required high-dose RT.

Different immune cell populations may exhibit intrinsic responsiveness to RT; however, the outcome on the TME depends on the recruitment of cells from the periphery, intra-tumoral proliferation, and the interplay of different cell populations responding to RT (8). While we found many changes to be tumor type dependent, we recognized common trends. For instance, macrophages, B cells, and DCs generally decreased (or were unchanged), whereas NK cells and monocytes (except for MC38-K tumors) typically increased. Granulocytes increased only in EMT6 tumors after intermediate-dose RT.

Optimizing RT dosing for anti-tumor immunity is challenging. Radiation was long considered immunosuppressive (74), in part because circulating CD8^+^ T cells may be among the most sensitive immune cells (74–76). However, even high-dose (ablative) local radiation requires CD8^+^ T cells for therapeutic efficacy (77, 78). At the same time, elective nodal irradiation can diminish the anti-tumor immune response induced by RT, likely by harming lymph-node resident CD8^+^ T cells (79, 80).

Our study focuses on the consequences of RT within the TME, including indirect effects on T cells such as recruitment from the periphery and egress. Because RT-induced cell death occurs relatively rapidly (≈2 days), our day-6 analysis is not suited to capture early depletion dynamics (81, 82). In models of low-dose RT that would be expected to preserve CD8^+^ T cells, irradiation reverses tumor desertification and enhances immunotherapy (83). However, in B16F10 tumors, 10 Gy recruited the highest number of CD8^+^ T cells among doses tested and promoted tumor vasculature normalization (84).

Cellular radiosensitivity is not uniform and Tregs are more resistant to RT than other T-cell subsets (85). In tumors, Tregs can increase after 10 Gy (86, 87), and memory CD8^+^ T cells are more resistant than naïve counterparts (88). Most importantly Arina et al. demonstrated that a substantial fraction of preexisting intra-tumoral T cells survived fractionated 5 × 1.8 Gy, single-dose 20 Gy, and even 30 Gy delivered as 10 + 20 Gy, maintaining motility and showing enhanced IFN-γ production via TME–driven reprogramming reminiscent of tissue-resident memory T cells (89). Together, these observations indicate that while RT can be immunosuppressive, it also induces and depends on anti-tumor T-cell responses, and key subsets (Tregs, memory, and especially tumor-resident CD8^+^ T cells) exhibit relative radio-resistance.

In our study, CD8^+^ T cells and Treg cells were notably increased only after intermediate-dose treatment, but not high-dose RT. In B16F10 tumors, the least RT-responsive model, CD8^+^ T cells decreased after RT compared with untreated tumors. Therefore, excessive RT dosing may impair anti-tumor T cell response, either directly through T cell death or indirectly by vascular damage or induction of immunosuppressive factors. These results highlight the importance of understanding the tumor type, TME and RT dose to achieve the desired therapeutic benefit.

Because TILs exhibit a high degree of complexity and diversity, we therefore performed scRNA-seq to gain deeper insight into the effects of RT on these dynamic cell states and types (90, 91). For monocyte and macrophage populations, those clusters with a proliferative signature were significantly reduced. With the notable exception again of B16F10 tumors, in which those clusters were unchanged, illustrating further the immunological resistance of B16F10 tumors to RT. We observed a strong increase in CD8^+^ T cells with a proliferative expression profile in all tumors except B16F10. This may help explain why B16F10 cancer cells, despite being sensitive to RT *in vitro*, are more resistant to RT *in vivo* compared to the other tumor types tested. Notably, overall CD8^+^ T cell infiltration (92, 93), and particularly the presence of T_PEX_ cells expressing TCF1 and PD-1, has been associated with favorable responses to CPI in melanoma patients (48, 94).

While our study does not distinguish whether the increased T cell numbers in the TME results from expansion of resident T cells or infiltration from the periphery. Prior studies suggest that immunotherapy largely depends on peripheral T cell recruitment (95, 96). Preclinical models have shown that sparing the TDLN enhances the anti-tumor efficacy of combined RT and immunotherapy (79, 80). Nevertheless, tumor-resident T cells can resist RT (89), and the anti-tumor effects of combined RT and anti-PD-1 therapy have been shown to involve both peripheral and resident T cell populations (97, 98). T_PEX_ cells can give rise to T_EX_ cells during CPI both in the tumor (48, 99) and in the TDLN (100–103). Both mechanisms may operate, as anti-tumor CD8^+^ T cells are activated in the TDLN but additional priming in the tumor to exert effector function (104).

One goal of this study was to understand the impact of RT on tumor models with different immunophenotypes and to develop a rationale-based framework for evaluating therapeutic mechanisms. Notably, the two MC38 tumor models differed in their monocyte population, which are precursors of macrophages and moDCs. MC38-R tumors contained more macrophages, whereas MC38-K tumors contained more monocytes. Interestingly, RT differentially affected monocyte, macrophage and DC fractions in MC38-R vs MC38-K models.

We hypothesized that these two models will exhibit different impacts on STING pathway/mechanisms. Activation of natural anti-tumor T-cell responses requires the cross-presentation of tumor-derived antigens by DCs to T cells (105). This process depends on IFN-I, which is essential for recruiting DCs to tumors and for their activation (106, 107). The cGAS-STING pathway plays a key role in this, as its activation triggers the secretion of IFN-I. This pathway is stimulated by the uptake of dying tumor cells and extracellular nucleosomes (108–111). Furthermore, the cGAS-STING pathway is essential for the efficacy of CPI (112–115). RT further amplifies the STING-mediated IFN-I production by enhancing the delivery of tumor DNA to DCs (116). We show that loss of STING activity in host cells leads to significant decrease in monocytes and increase in macrophages in MC38-K tumors after RT, while it has the opposite effect in MC38-R tumors. Previous reports showed that monocytes invading MC38-R tumors are responsible for blunting the anti-tumor immune response induced by RT (58). We sought to investigate the effect of STING deficiency in MC38-K tumors, a model characterized by high genetic similarity but distinguished by a significantly larger population of monocytes expressing an IFN-I response signature. In this model, we found that RT not only failed to increase the fraction of monocytes but instead resulted in its reduction, while concurrently increasing the fraction of macrophages. Notably, STING deficiency did not result in reduced TGI in MC38-R tumors, likely due to the compensatory recruitment of immunosuppressive monocytes in the presence of STING (59). However, STING was essential for the TGI observed in MC38-K tumors. STING plays a context-dependent role in TGI, being essential in MC38-K tumors with an IFN-I response signature, but dispensable in MC38-R tumors where compensatory immunosuppressive mechanisms predominate.

Recent work has contrasted shielded non-conformal RT (SRT), the common preclinical approach, with clinically relevant conformal RT (CRT). CRT spared normal tissue and delivered as 20 Gy single fraction or 3 × 8 Gy rapidly recruited pro-inflammatory monocytes to MC38 tumors (117). In contrast, SRT recruited fewer monocytes that preferentially differentiated into TAM-like suppressive cells. IFN-I signaling in monocytes was indispensable for CRT efficacy. Global Ifnar1^-/-^ mice failed to control tumors after CRT or SRT. When only monocytes were IFNAR-deficient, CRT efficacy and CD8^+^ TIL function were impaired, whereas T cell–restricted IFNAR deficiency caused only partial loss of activity. Notably, acute IFN-I induction after CRT was STING-independent. Our work adds to these findings that, in addition to the RT delivery mode, the TME also influences the type of monocytes infiltrating the tumor independent of treatment. We can speculate, based on the findings of Tadepalli et al. (117), that IFNAR deficiency in monocytes might compromise the anti-tumor benefit of RT. In accordance with the findings of Tadepalli et al. (117), the effect in our SRT treatment is STING-dependent.

Kinases are attractive drug targets due to the druggable nature of their ATP-binding domains and the demonstrated clinical success of kinase inhibitors in treating cancer. HPK1, a kinase that negatively regulates T cell activation, has emerged as a promising target for immunotherapy (118). Small molecule drugs offer advantages such as oral bioavailability for easier administration, lower manufacturing costs, and shorter half-lives that allow for precise dosing and reduced risk of prolonged adverse effects. Because we observed an increase in CD8^+^ T cells following RT, we tested the effects of RT in an HPK1 deficient background. Indeed, tumor-targeted RT in HPK1-deficient mice lead to a significant increase in CD8^+^ T cells, as well as significant increase in survival. HPK1 inhibition has been widely implicated in promoting anti-tumor immunity. While our data are limited, they raise the possibility that combining HPK1 inhibitors with RT may further augment immune-mediated tumor control.

Our study has several limitations that should be considered when interpreting the findings. First, although we tested three radiation doses that span a broad range, additional regimens could reveal further effects. For example, fractionated schedules such as 3 × 6 Gy, or alternative dosing schemes, may influence immune responses in ways not captured here. Second, we focused our analyses on specific post-treatment time points. While day 6 was chosen as a biologically meaningful window that allows immune responses to develop, we cannot exclude that some immune populations may undergo transient depletion immediately after irradiation before being replenished by peripheral recruitment. Finally, our study provides limited mechanistic insight. The primary objective was to generate a dataset that enables rationalistic development of radiation–immunotherapy combinations. Further mechanistic studies and combination therapies with pathway inhibitors or activators will be needed to delineate the underlying cellular and molecular pathways. It will also be interesting to compare the effects of radioligand therapy with alpha- and beta-radiation on TME (119).

In summary, our data suggest that the immune consequences of RT are context-dependent, varying across tumor types and RT doses in relation to the immune composition of the TME. Supporting this, studies in STING- and HPK1-deficient mice showed that the benefit of combining RT with immune modulation depends on the immune context at the time of treatment. It will be interesting in the future to evaluate the rationalistic combination strategy with RT using small molecule or biologic therapies, novel or approved in this setting.

## Data Availability

The scRNA Seq data presented in the study are deposited in the GEO repository and the accession number is GSE294347.

## Supplementary material

Supplementary Figure 1 Gating strategy for flow cytometry analysis of **(A)** splenocytes, and **(B)** tumor draining lymph node cells. Single-cell suspensions were prepared from spleens and lymph nodes. Cells were stained with fluorochrome-conjugated antibodies against surface and intracellular markers as indicated. Data were acquired on a BD LSRFortessa X-20 flow cytometer and plotted using FlowJo 10 software. Red arrows indicate sub-gating. DC, dendritic cell; NK T, Natural Killer T cell; NK, Natural Killer cell; B, B cell; GC B, Germinal center B cell; γδ T, Gamma Delta T cell; CD8 T, CD8^+^ T cell; CD4 T, CD4 T cell; Treg, regulatory T cell; Gran, Granulocyte; Mono, Monocyte; MΦ, Macrophage; * for BALB/c animals used NKp46.

Supplementary Figure 2 Quantification of immune cell infiltration across syngeneic tumor models (CT26, MC38-R, MC38-K, EMT6 and B16F10). Mice were implanted with cancer cells in the right flank, and tumors were harvested 15–21 days post-implantation. Tumors were processed into single-cell suspensions and analyzed by flow cytometry. **(A–I)** Absolute number (cells per mg tumor tissue) of infiltrated CD45^+^ cells, macrophages, monocytes, granulocytes, CD8^+^ T cells, Tregs, B cells, dendritic cells, and NK cells, respectively. Note that CD45^+^ graph **(A)** is duplicate of **Figure 1A** and is used here for easier comparison with other TILs. Bars represent mean ± SD (N = 4–6 mice per group).

Supplementary Figure 3 Biodistribution (BioD) score calculation from IHC images of tumors. **(A)** Steps involved in the calculation of the BioD score from an IHC image. The tumor area is divided into ten annular zones of equal area and the average CD8α cell density in each zone is computed. Annular zones are then binned into 3 regions, i.e., core, intermediate and periphery and the average CD8a cell density in each region is calculated. The BioD score is then given by the pairwise ratio of the average CD8α cell density in the different regions. **(B)** Simulated examples of different CD8α spatial distribution patterns (black dots). For each scenario, core, intermediate and periphery regions are shown and BioD score is calculated. For comparison, the tissue size and shape are kept identical in all the three scenarios.

Supplementary Figure 4 The proportion of exhausted CD8^+^ T (TEX) cells (PD-1^+^TOX^+^) did not increase following RT. Mice were implanted with syngeneic cancer cells and treated with the indicated dose of RT. Six days after RT, tumor weights were measured **(A)** and TILs were analyzed by flow cytometry **(B)**; ± SD is shown, N = 6–8 samples/group.

Supplementary Figure 5 RT leads to increased CD8α and FoxP3 staining in CT26 and MC38 tumors. **(A)** Representative microscopy images showing immunohistochemistry staining for CD8α. **(B)** Representative microscopy images showing immunohistochemistry staining for FoxP3. (N = 4-6). Scale bar – 75 µm.

Supplementary Figure 6 Immunological changes in spleen after tumor-targeted RT. Mice were implanted with tumor cells and treated with the indicated dose of RT. Six days after RT (12 Gy or 2×12 Gy dose), splenocytes were analyzed by flow cytometry. Changes in different immune cell population in spleen is shown here: **(A)** B cells, **(B)** CD4^+^ T cells, **(C**) CD8^+^ T cells, **(D)** NK cells, **(E)** Tregs, **(F)** Granulocytes, **(G)** Eosinophils, **(H)** Monocytes, **(I)** Macrophages, **(J)** Ly6C- Monocytes. Asterisks denote significance: *p < 0.05, **p < 0.01, ***p < 0.001, and ns - not significant. Bars show average % of cells along with ± SEM (N = 4–6).

Supplementary Figure 7 Immunological changes in TDLN cells after tumor-targeted RT. Mice were implanted with syngeneic cancer cells and treated with the indicated dose of RT (12 Gy or 2×12 Gy dose). Six days after RT, inguinal lymph nodes were analyzed by flow cytometry. Changes in different immune cell population in lymph nodes are shown here: **(A)** T cells, **(B)** CD4^+^ T cells, **(C)** CD8^+^ T cells, **(D)** B cells, **(E)** GC cells, **(F)** NK cells, **(G)** NK T cells. Asterisks denote significance: *p < 0.05, **p < 0.01, ***p < 0.001, and ns - not significant. Bars show average % of cells along with ± SEM (N = 4-6).

Supplementary Figure 8 Heterogeneous effects of RT on additional T-cell clusters across tumor models. Mice were implanted with syngeneic cancer cells and treated with the indicated dose of RT. Six days after RT, TILs were analyzed by flow cytometry. Changes in the abundance of T_PEX_**(A)**, T_EX_**(B)**, and _TEFF_**(C)** T-cell clusters following RT were assessed across tumor models. Pairwise comparisons 12 Gy vs 0 Gy across cell populations and mouse models were performed using t-tests. Asterisks denote significance: *p < 0.05, **p < 0.01, ***p < 0.001, and ns - not significant; ± SD is shown, N = 3–4/group.

Supplementary Table 1 Flow cytometry antibodies. Antibodies used for flow cytometry, including fluorophore, target antigen, clone, catalog number, and vendor.

Supplementary Table 2 Summary statistics for the RT treatment effects on tumor growth and immune populations across syngeneic tumor models. Summary statistics for (i) tumor growth inhibition (TGI), (ii) flow cytometry–defined CD8^+^ T-cell and NK-cell frequencies, and (iii) single-cell RNA-seq–defined proliferating macrophage and CD8 T-cell clusters are reported across models and treatments. Treatment effects on tumor growth are summarized as tumor change ratios with derived TGIs. Immune readouts are summarized as ratios of population frequencies. The 95% confidence intervals, p-values, and additional information related to the statistical tests applied are also reported.

Supplementary Table 3 Correlation of tumor growth inhibition and *in vitro* confluency with baseline CD8^+^ T-cell numbers and BioD C:P scores across tumor models. Correlation analyses were performed between *in vivo* tumor growth inhibition (TGI) and *in vitro* confluency across tumor models with baseline CD8^+^ T-cell numbers (flow cytometry) and BioD C:P scores. Analyses were stratified by RT dose–adjusted, 6 Gy, and 2 × 12 Gy treatment groups. Sample sizes were N = 4–10 per group for *in vivo* studies and N = 2 for the *in vitro* study.

Supplementary Table 4 Differentially expressed genes for each cluster found in the tSNE plot. Different sheets represent different tumor models - 132 is MC38; 145 is CT26; 146 is B16F10; 161M is EMT6. M and T is monocytes/macrophages and T cells, respectively.
