# Supplementary material for: Rational modulation of immune mechanisms synergizes the anti-tumor effects of targeted radiation therapy in pre-clinical models
Source: Front Immunol. 2026 Mar 27;17:1637129. doi: 10.3389/fimmu.2026.1637129 (PMC13081731; doi:10.3389/fimmu.2026.1637129)

Supplementary Figure 1A

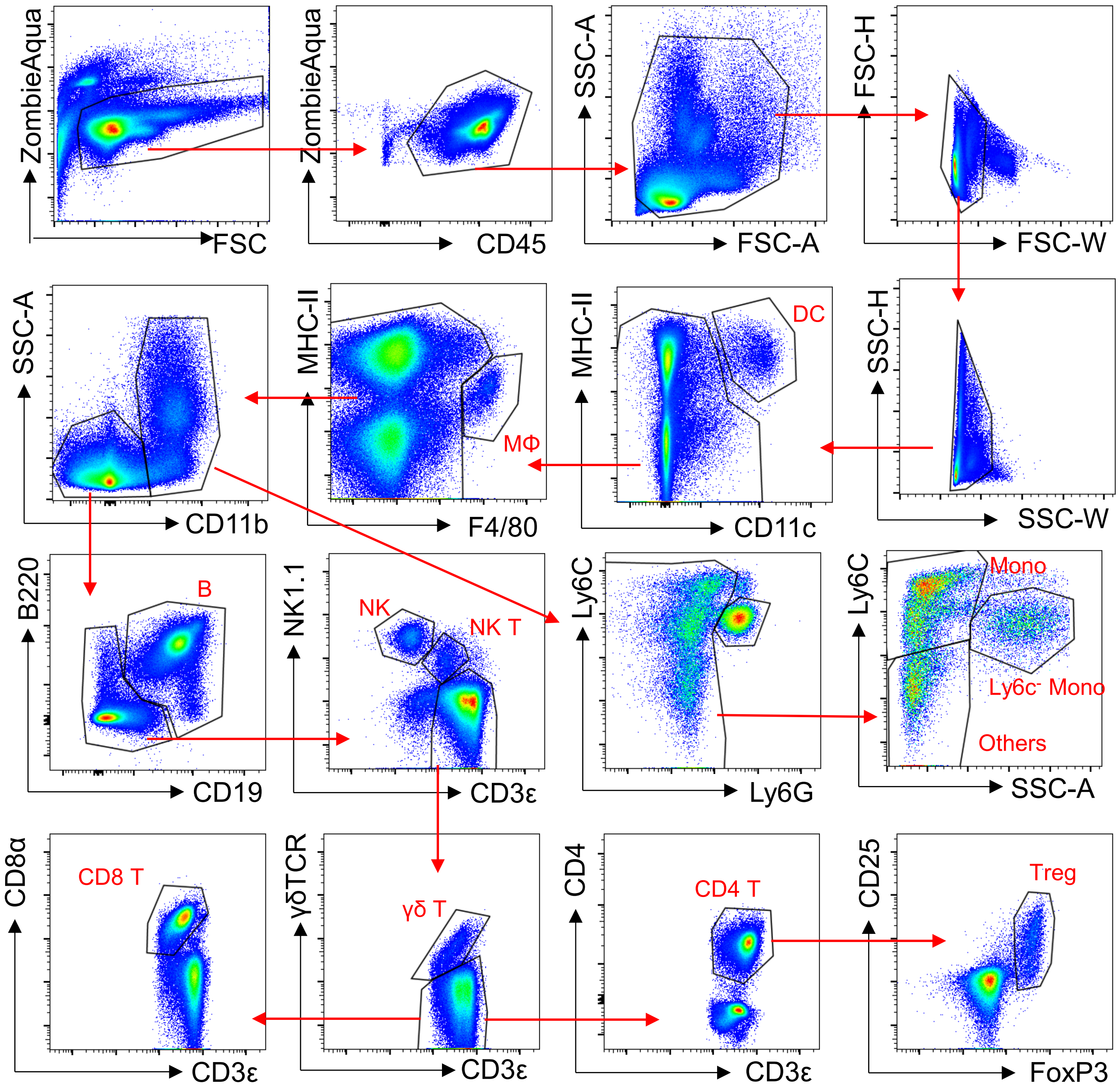

Supplementary Figure 1B

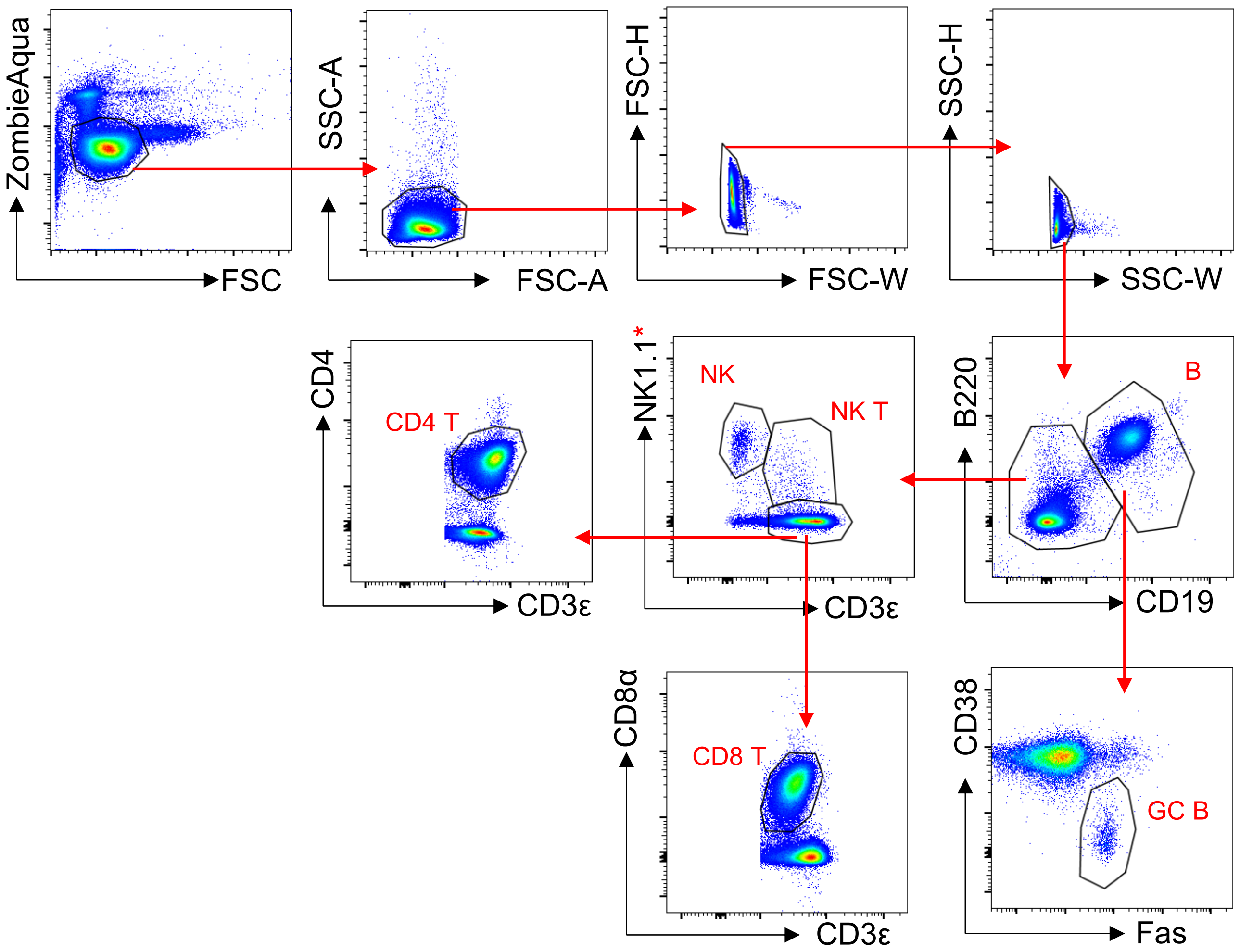

Supplementary Figure 2

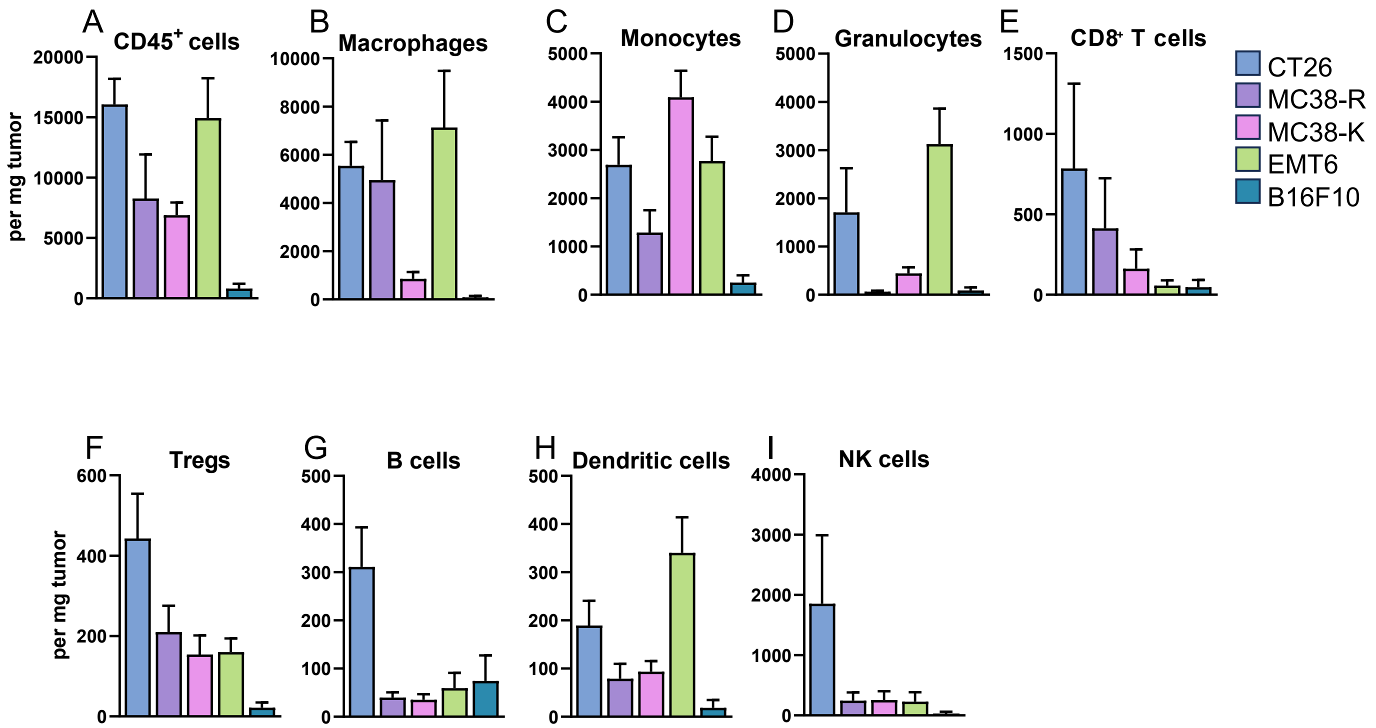

Supplementary Figure 3

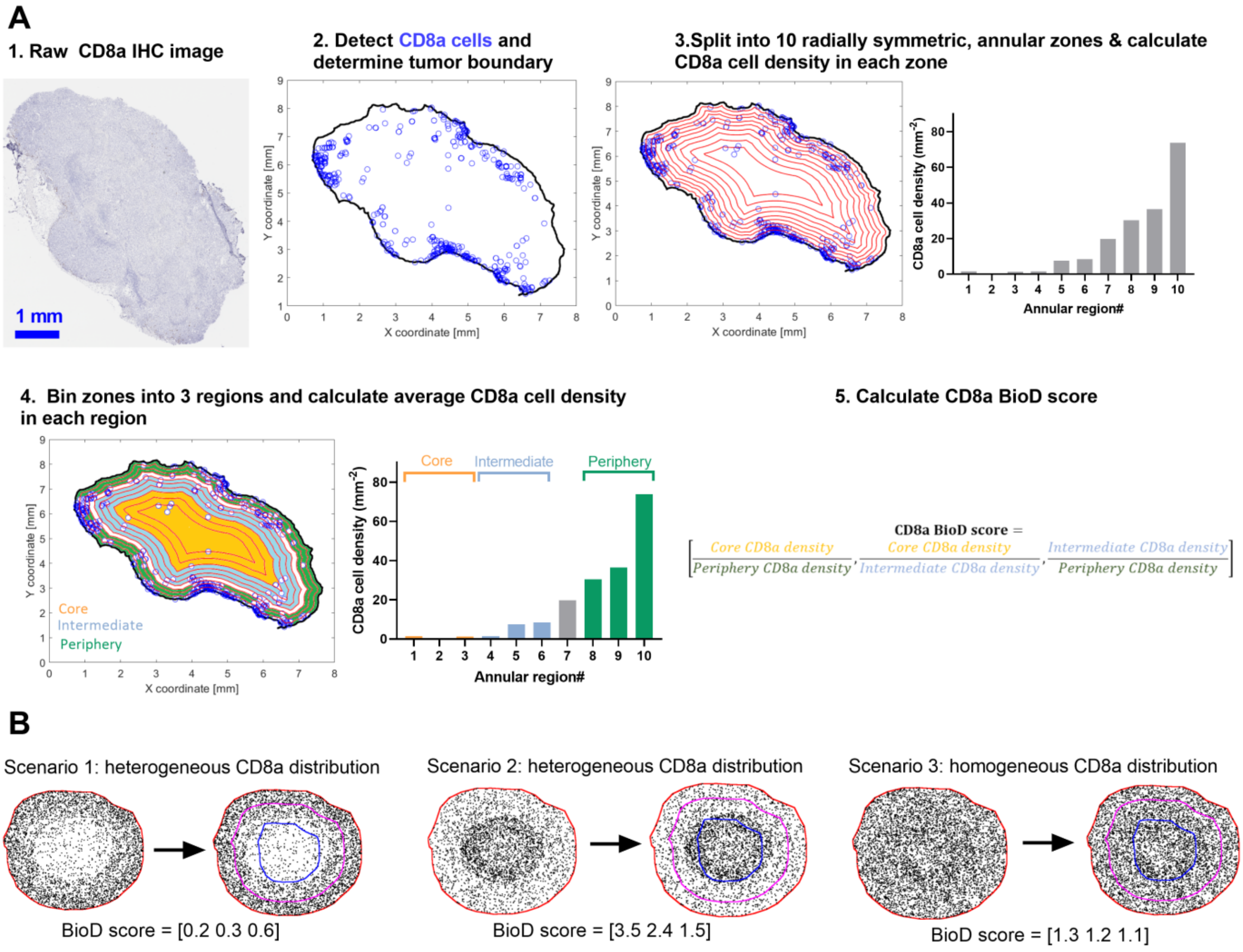

Supplementary Figure 4

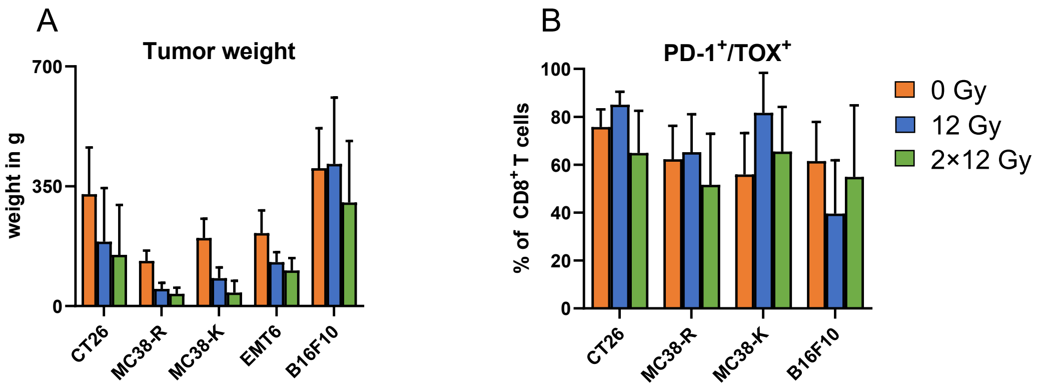

Supplementary Figure 5

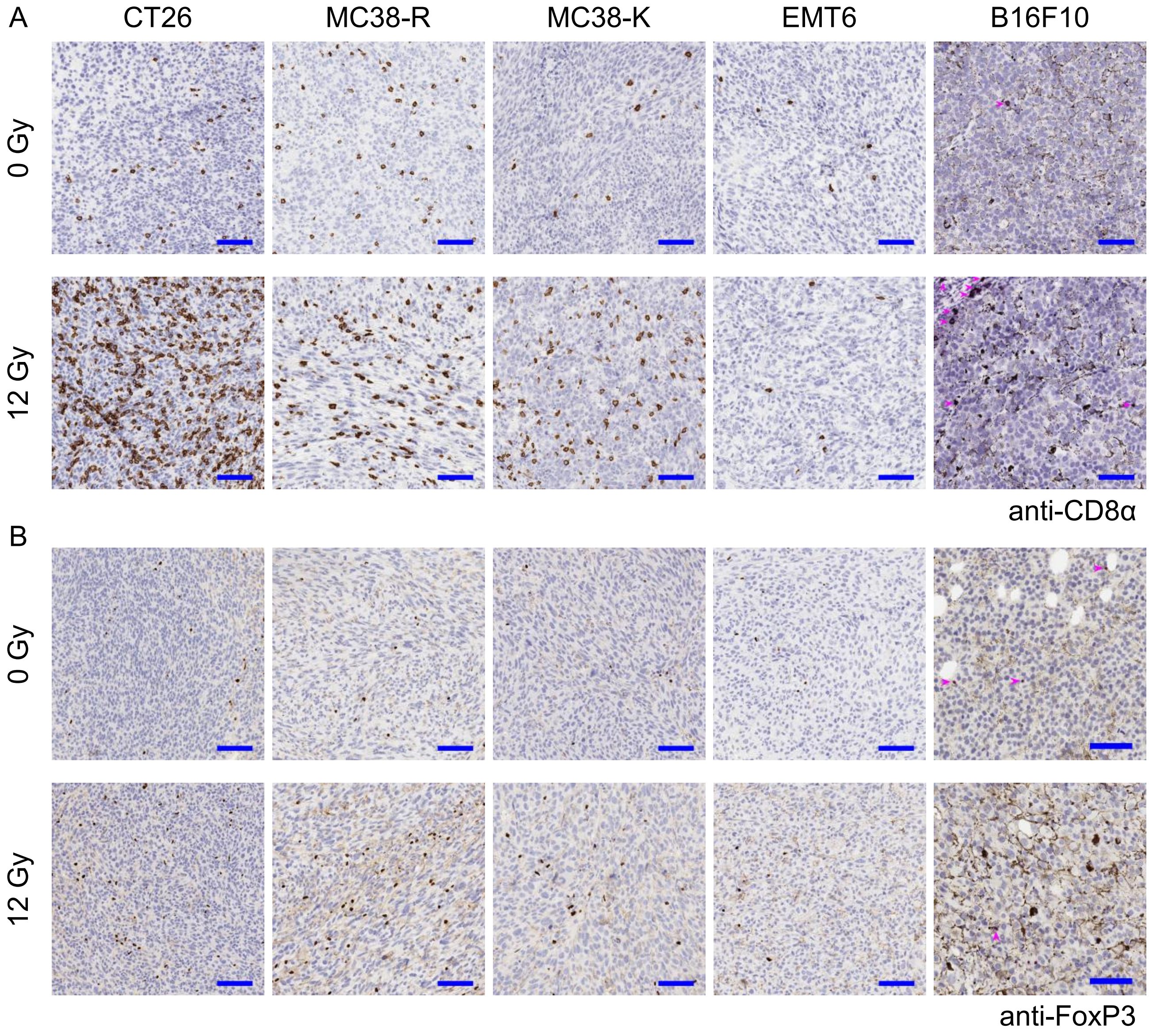

Supplementary Figure 6

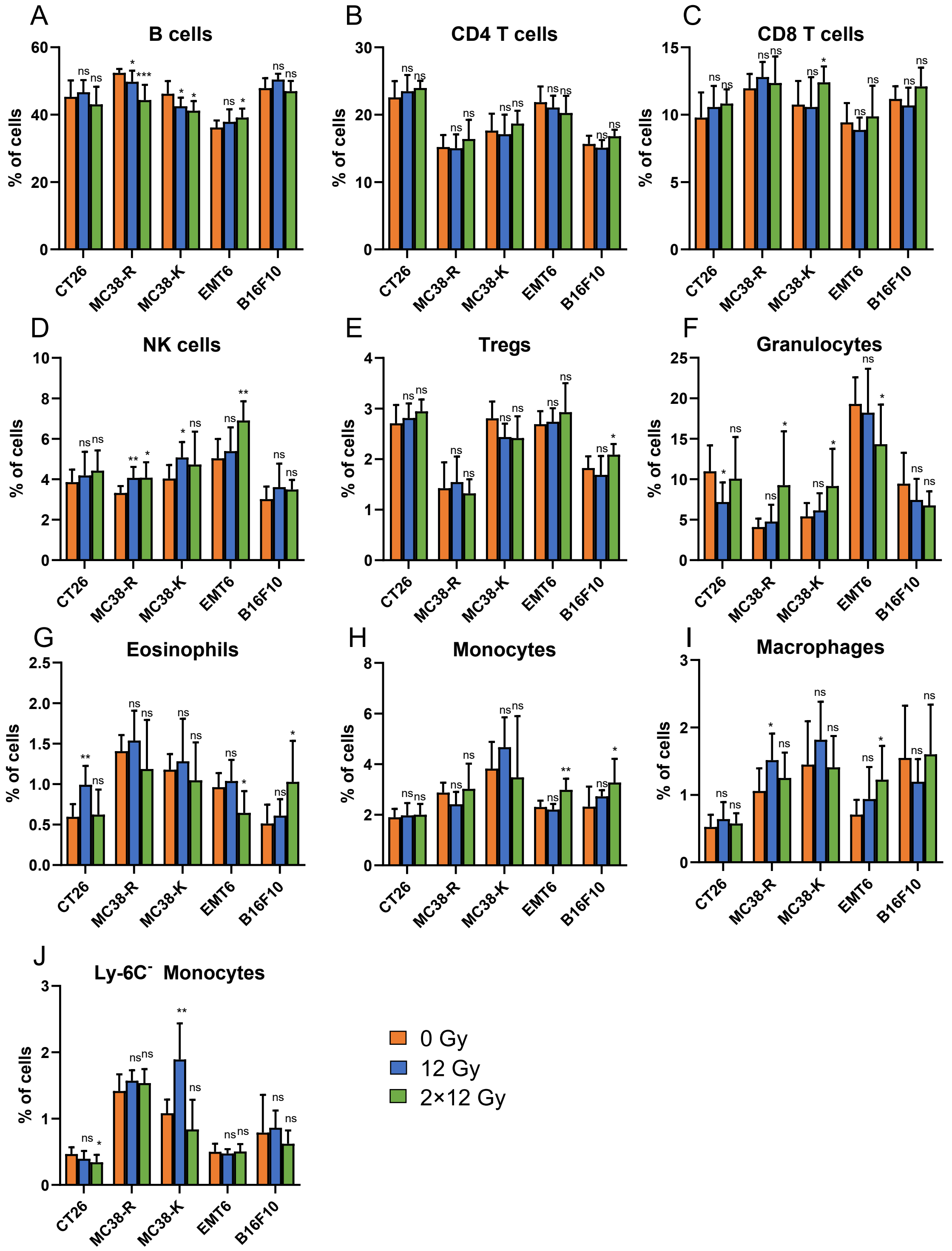

Supplementary Figure 7

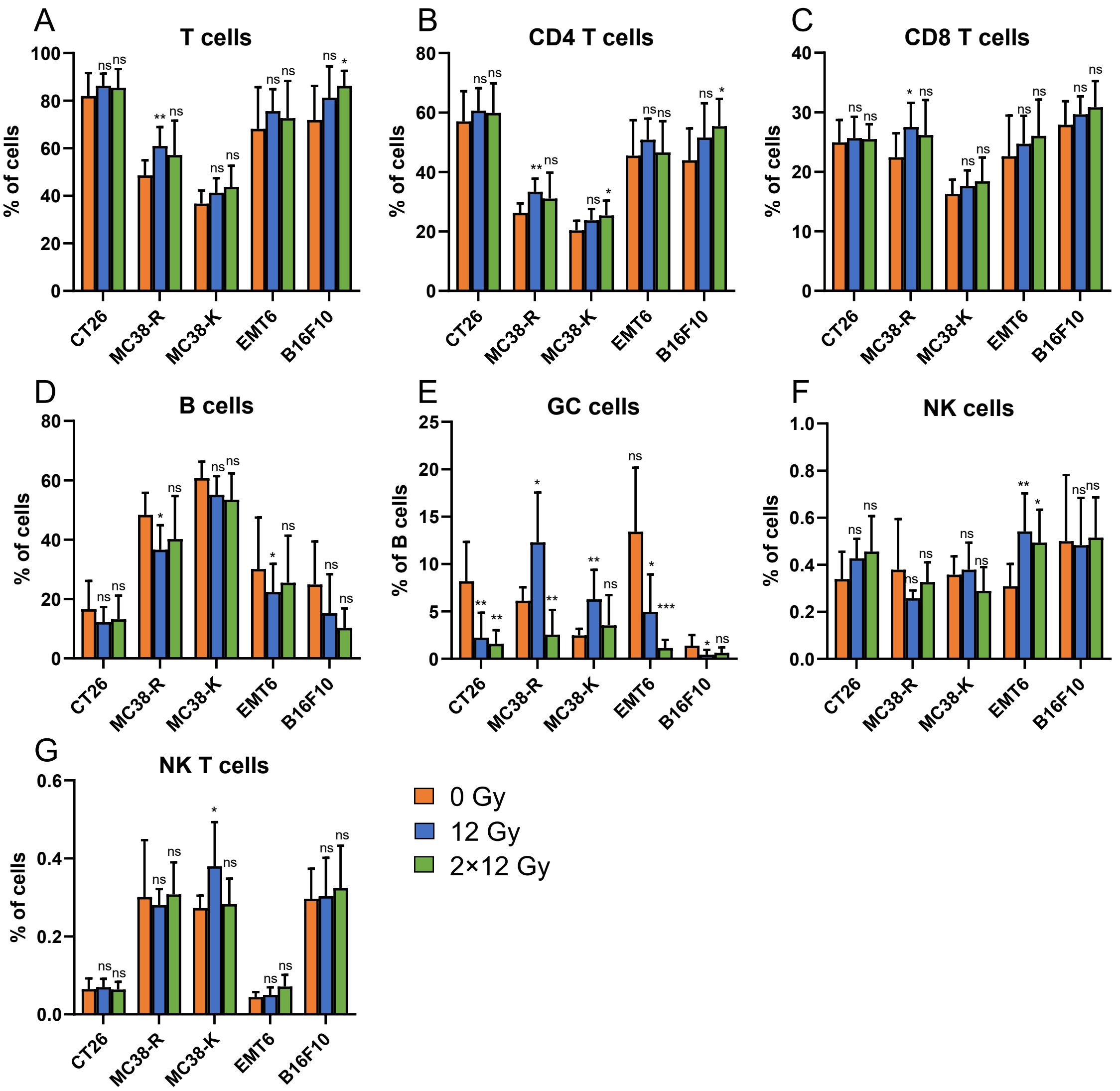

Supplementary Figure 8

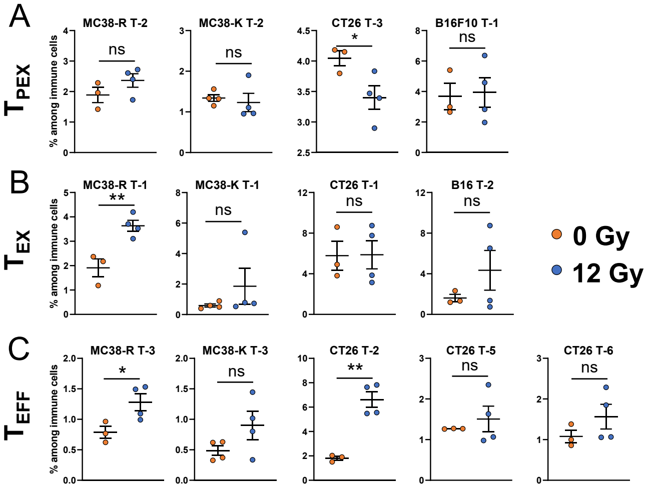

Supplement: Supplementary Figure 1 — Gating strategy for flow cytometry analysis of (A) splenocytes, and (B) tumor draining lymph node cells. Single-cell suspensions were prepared from spleens and lymph nodes. Cells were stained with fluorochrome-conjugated antibodies against surface and intracellular markers as indicated. Data were acquired on a BD LSRFortessa X-20 flow cytometer and plotted using FlowJo 10 software. Red arrows indicate sub-gating. DC, dendritic cell; NK T, Natural Killer T cell; NK, Natural Killer cell; B, B cell; GC B, Germinal center B cell; γδ T, Gamma Delta T cell; CD8 T, CD8+ T cell; CD4 T, CD4 T cell; Treg, regulatory T cell; Gran, Granulocyte; Mono, Monocyte; MΦ, Macrophage; * for BALB/c animals used NKp46. [file DataSheet1.pdf]
