# Supplementary material for: Rational modulation of immune mechanisms synergizes the anti-tumor effects of targeted radiation therapy in pre-clinical models
Source: Front Immunol. 2026 Mar 27;17:1637129. doi: 10.3389/fimmu.2026.1637129 (PMC13081731; doi:10.3389/fimmu.2026.1637129)
Supplement: Supplementary Table 1 — Flow cytometry antibodies. Antibodies used for flow cytometry, including fluorophore, target antigen, clone, catalog number, and vendor. [file Table1.docx]

**Supplementary Table 1.** List of antibodies and staining reagents for flow cytometry.

| **Fluorophore** | **Antigen** | **Clone** | **Catalog Number** | **Vendor** |
| --- | --- | --- | --- | --- |
| AF488 | CD3ε | 145-2C11 | 557666 | BD |
| FITC | CD3ε | 145-2C11 | 553062 | BD |
| PerCP-Cy5.5 | Ly-6C | HK1.4 | 128012 | Biolegend |
| APC | CD11c | N418 | 117310 | Biolegend |
| AF647 | CD11c | N418 | 117312 | Biolegend |
| Alexa700 | I-A/I-E | M5/114.15.2 | 107622 | Biolegend |
| APC-Cy7 | CD11b | M1/70 | 101226 | Biolegend |
| PE | F4/80 | BM8 | 123110 | Biolegend |
| Zombie Aqua™ Fixable Viability Kit |  |  | 423102 | Biolegend |
| PE-Cy7 | CD64 | X54-5/7.1 | 139323 | Biolegend |
| BUV395 | CD45 | 104 | 741957 | BD |
| BUV496 | CD4 | GK1.5 | 612952 | BD |
| BUV737 | CD19 | 6D5 | 115537 | Biolegend |
| BUV805 | CD8α | 53-6.7 | 612898 | BD |
| BV421 | NK1.1 | PK136 | 108741 | Biolegend |
| BV421 | NKp46 | 29A1.4 | 562850 | BD |
| BV510 | Zombie Aqua |  |  |  |
| BV605 | γδ TCR | GL3 | 118129 | Biolegend |
| BV650 | Ly-6G | 1A8 | 740554 | BD |
| BV785 | B220 | RA3-6B2 | 103246 | Biolegend |
|  | TruStain FcX | 93 | 101320 | Biolegend |
| APC | CD38 | 90 | 102712 | Biolegend |
| AF647 | CD38 | 90 | 102716 | Biolegend |
| PE-Cy7 | Fas | Jo2 | 557653 | BD |
| PerCP-Cy5.5 | FOXP3 | FJK-16s | 45-5773-82 | eBioscience |
| Alexa700 | Ki-67 | SolA15 | 56-5698-82 | eBioscience |
| PE | TOX | TXRX10 | 12-6502-82 | eBioscience |
| BV421 | CD25 | PC61 | 102043 | Biolegend |
| BV605 | PD-1 | 29F.1A12 | 135220 | Biolegend |
