# Supplementary material for: Rational modulation of immune mechanisms synergizes the anti-tumor effects of targeted radiation therapy in pre-clinical models
Source: Front Immunol. 2026 Mar 27;17:1637129. doi: 10.3389/fimmu.2026.1637129 (PMC13081731; doi:10.3389/fimmu.2026.1637129)
Supplement: Supplementary Table 3 — Correlation of tumor growth inhibition and in vitro confluency with baseline CD8+ T-cell numbers and BioD C:P scores across tumor models. Correlation analyses were performed between in vivo tumor growth inhibition (TGI) and in vitro confluency across tumor models with baseline CD8+ T-cell numbers (flow cytometry) and BioD C:P scores. Analyses were stratified by RT dose–adjusted, 6 Gy, and 2 × 12 Gy treatment groups. Sample sizes were N = 4–10 per group for in vivo studies and N = 2 for the in vitro study. [file Table3.docx]

**Supplementary Table 3.** Correlation Analysis *in vivo* TGI and *in vitro* confluency across tumor models with CD8⁺ T cell number at enrollment as determined by flow cytometry and BioD C:P scores. There was a good correlation between *in vivo* TGI with CD8⁺ T cell number and BioD scores in RT dose-adjusted, 6 Gy and 2 X 12 Gy groups. As anticipated, there was no correlation between CD8 T cell number or BioD scores when compared with % confluency. N = 4-10/group (*in vivo* studies); N = 2 (*in vitro* study).

**Spearman correlations with *in vivo* TGI**

|  | CD8⁺ T cell | BioD C:P score |
| --- | --- | --- |
| 6 Gy | 1 | 1 |
| 12 Gy | 0.3 | -0.4 |
| 2 X 12 Gy | 0.9 | 0.8 |
| Dose-adjusted | 0.73 | 0.61 |
| **Spearman correlations with Slope of *in vitro* confluency curves** | | |
|  | CD8⁺ T cell | BioD C:P score |
| 0 Gy | -0.80 | -0.83 |
| 3 Gy | 0.10 | -0.80 |
| 6 Gy | 0.00 | -1.00 |
| 12 Gy | 0.10 | 0.53 |
| Dose-adjusted | -0.15 | -0.55 |
